# Supplementary material for: Dormancy heterogeneity among Arabidopsis thaliana seeds is linked to individual seed size
Source: Plant Commun. 2023 Oct 12;5(2):100732. doi: 10.1016/j.xplc.2023.100732 (PMC10873894; doi:10.1016/j.xplc.2023.100732)
Supplement: Document S1. Supplemental Figures 1–8 [file mmc1.pdf]

Plant Communications, Volume *5*

Supplemental information

Dormancy heterogeneity among *Arabidopsis thaliana* seeds is linked to individual seed size

Michal Krzyszton, Sebastian P. Sacharowski, Veena Halale Manjunath, Katarzyna Muter, Grzegorz Bokota, Ce Wang, Dariusz Plewczyński, Tereza Dobisova, and Szymon Swiezewski

Supplemental Figures

Dormancy heterogeneity among *Arabidopsis thaliana* seeds is linked to individual seed size

Michal Krzyszton, Sebastian P. Sacharowski, Veena Halale Manjunath, Katarzyna Muter, Grzegorz Bokota, Ce Wang, Dariusz Plewczyński, Tereza Dobisova, Szymon Swiezewski

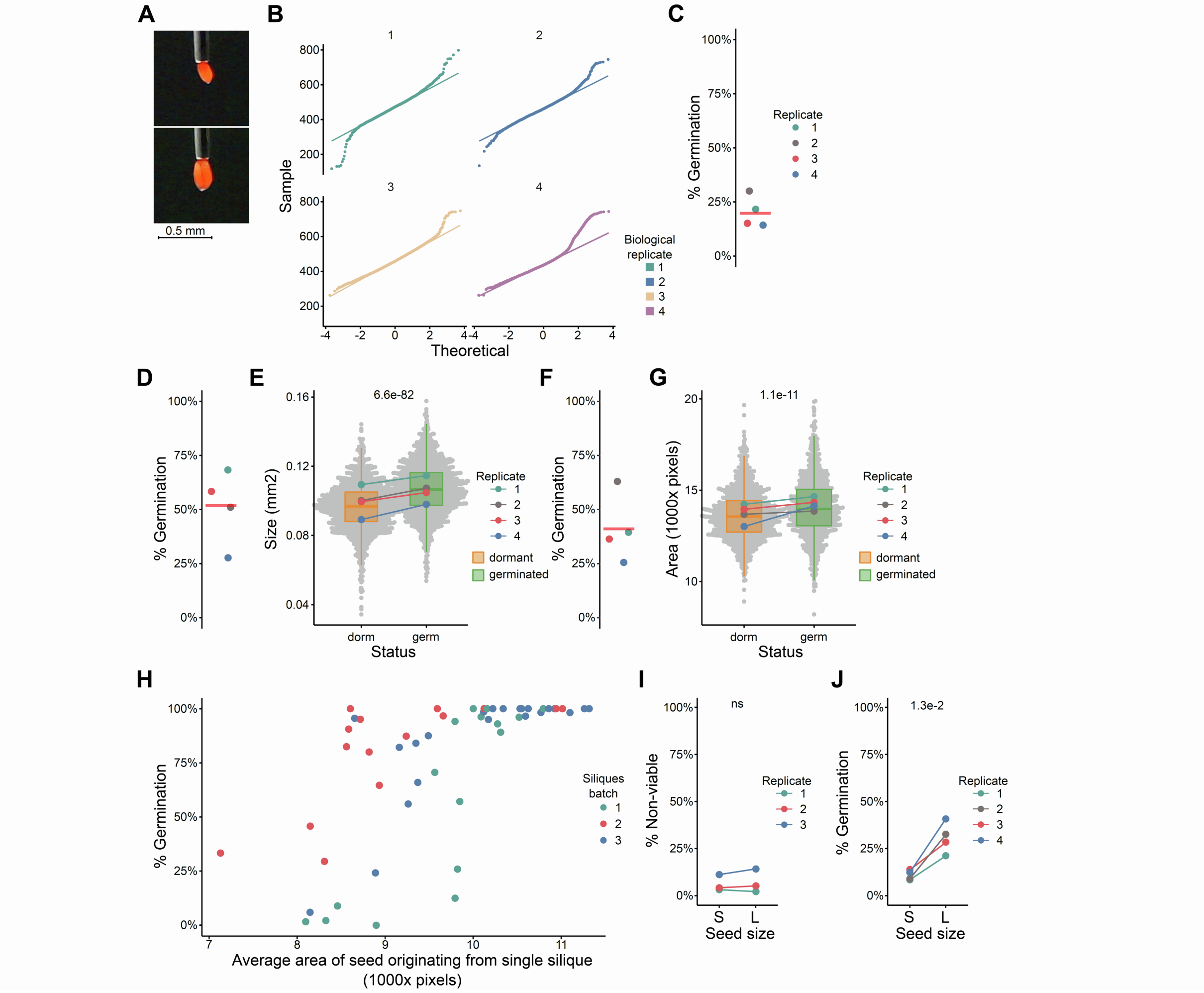

Supplemental Figure 1. Small seeds are more dormant. A) Examples of seeds' pictures obtained using the Boxeed robot. B) Deviations from the normal distribution of seed sizes in four seed pools from Figure 1A are shown using Q-Q plots. C) Germination of freshly harvested Col-0 seeds (n=320 to 607) was analyzed in four replicas. The red line denotes the mean value. D) Germination of after-ripened Col-0 seeds after 7 days of secondary dormancy induction (n=735 to 808) was analyzed in four replicas. The red line denotes the mean value. E) Size distribution of germinated (germ) and dormant (dorm) seeds from D. Mean values of seed sizes for dormant and germinated seeds are shown as points for each of the replicates. The p-value of the Wilcoxon rank-sum test for the comparison of seed sizes from all replicas is shown above the plot. F) Germination of Col-0 after-ripened seeds after 7 days of secondary dormancy induction. Four replicas of seed pools (n=450 to 799) were placed on the plate, photographed using a stereo microscope and seed sizes were assayed using ImageJ software. The red line denotes the mean value. G) Size distribution of germinated (germ) and dormant (dorm) seeds from F. Mean values of seed sizes for dormant and germinated seeds are shown as points for each of the replicas. The p-value of the Wilcoxon rank-sum test for seed sizes from all replicas is shown above the plot. H) Germination percentages of seeds from each silique were plotted against the average size of seeds from the siliques. I) Percentage of non-viable seeds after secondary dormancy induction treatment. Plates after scoring initial germination were placed for 7 days at 4°C after which seeds were again scored for seed germination. Seeds that did not germinate were considered non-viable. "ns" marks a lack of statistical significance (two-sided

paired t-test). J) The top 10% of the smallest and the largest seeds (n= 116-211) from four Col-0 after-ripened seed pools were sorted and tested for germination in the presence of 0.5 mM ABA. The p-value of the two-sided paired t-test is shown above the plot. Seeds were analyzed using a Boxeed robot. Seed germination was scored after 7 days.

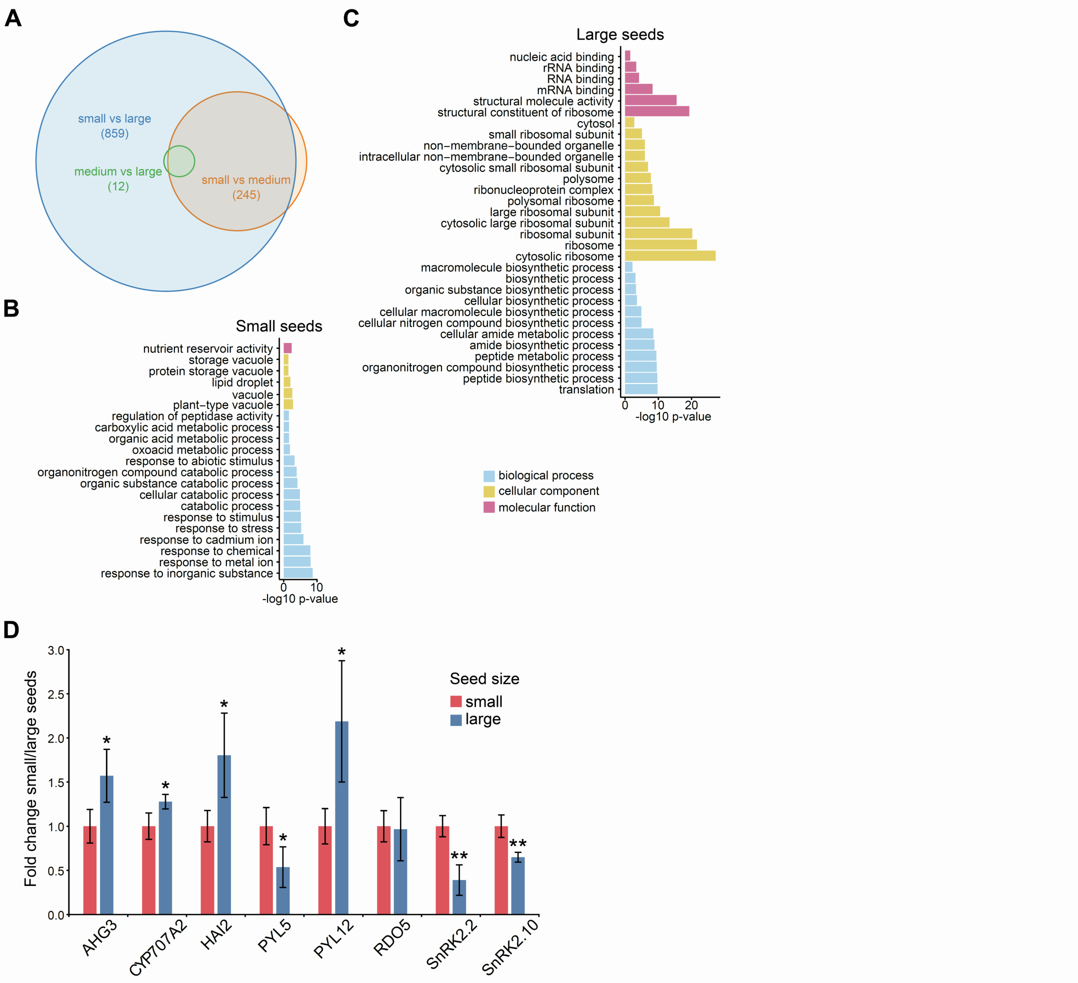

Supplemental Figure 2. Small and large seeds differ in transcriptome profiles. A) Differential gene expression analysis reveals that a similar set of genes is affected (DESeq2, padj < 0.05) when small, medium, and large seeds are compared. B) and C) GO terms enriched (gprofiler2) among genes with higher expression in small and large seeds, respectively. Colors show sources of gene ontologies. D) RT-qPCR analysis (four replicas of large and small Col-0 seeds) of selected genes with expression change in 3′RNA-seq. Fold change to the average value for small seeds is shown for each transcript and error bars show the standard deviation. Asterisks denote statistical significance (t-test): * p-value < 0.05 and ** p-value < 0.01. *UBC21* mRNA was used as a reference.

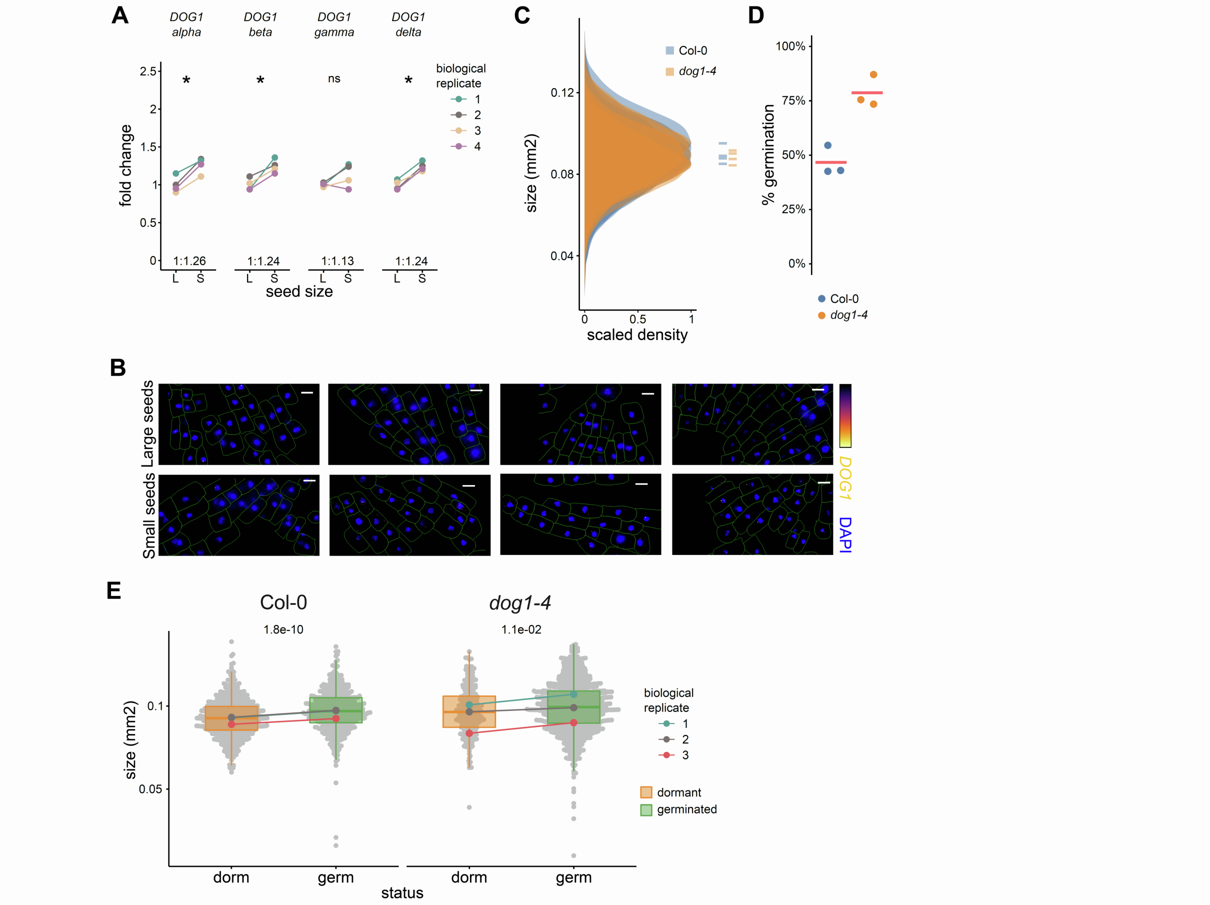

Supplemental Figure 3. *DOG1* expression differentiates seeds of different sizes. A) RT-qPCR analysis of long *DOG1* splicing isoforms (*alpha*, *beta*, *gamma*, *delta*) in four replicas of large and small Col-0 seeds. Fold change to the average value for large seeds is shown for each transcript. Asterisks denote p-value < 0.05, and "ns" marks a lack of statistical significance (paired t-test). Ratios of isoform levels are shown above the x-axis. *UBC21* mRNA was used as a reference. B) z-stack max-projection images of smFISH for *DOG1* mRNA. The "Inferno" colour scale is used for the intensity of fluorescence from Quasar670 fluorophore (*DOG1*). The blue colour shows *DAPI* fluorescence from (nuclei staining). The scale bar is 20 µm. C) Seeds' sizes of Col-0 and *dog1-4* mutant were obtained using the Boxeed robot. Median values of seeds' size for each tested seed pool are shown as a line on the right. 3000 seeds were analyzed for each pool. D) Col-0 and *dog1-4* mutant after-ripened seeds germination after 7 days of secondary dormancy induction (n=354 to 384). Red lines denote mean values. E) Size distribution of germinated (germ) and dormant (dor) seeds from D. Mean values of seed sizes for dormant and germinated seeds are shown as points for each of the replicas. The p-values of the Wilcoxon rank-sum test for comparison of seed sizes for all replicas are shown above the plots. Seeds were sown using a Boxeed robot and seeds' morphological parameters were obtained using Boxeed software.

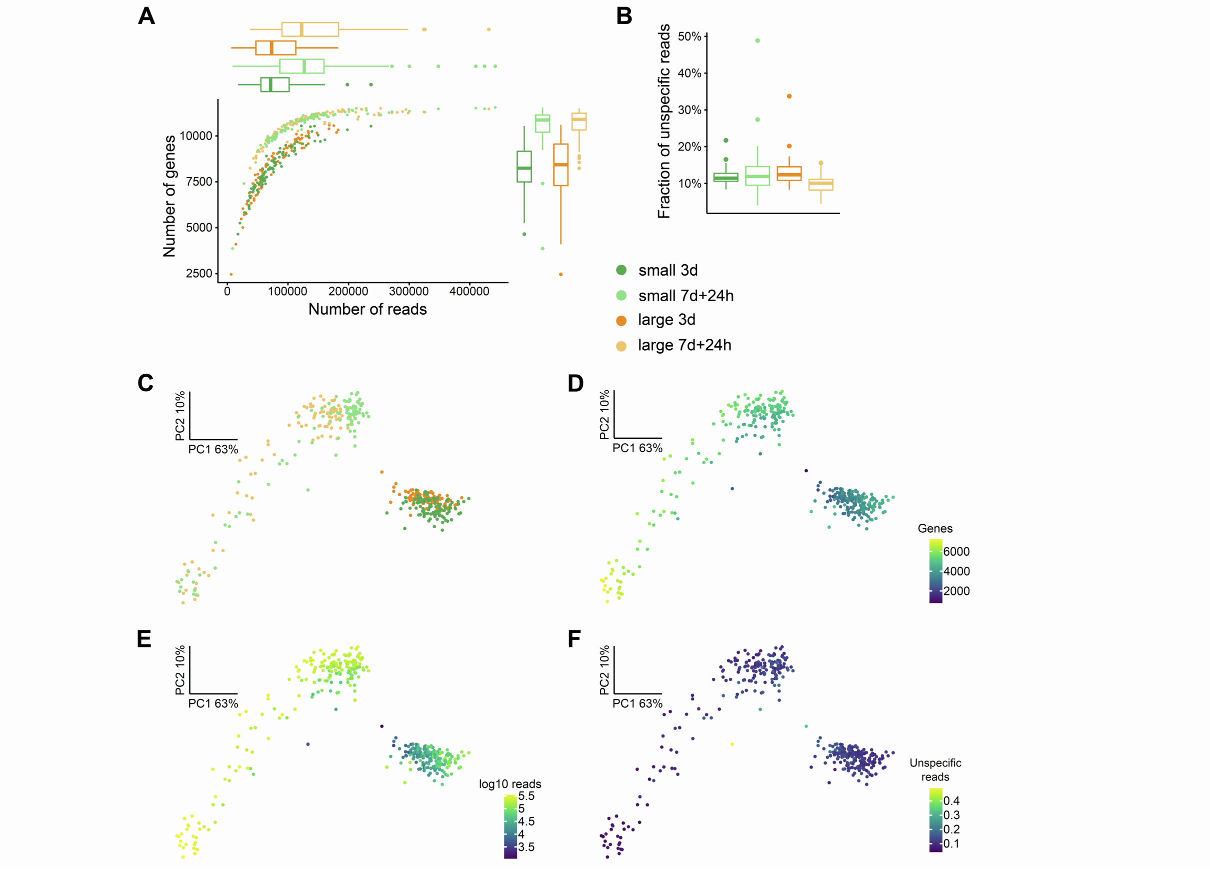

Supplemental Figure 4. Quality controls for single seed RNA-seq from small and large seeds experiment. A) Plot showing the number of reads sequenced and genes identified for each seed (n = 384). Boxplots above and on the right of the plot show the number of reads sequenced and genes identified respectively for each time point. Boxplot whiskers show a 1.5 interquartile range, outliers are marked with dots. B) Boxplots showing the fraction of unspecific reads for each seed in each condition. Boxplot whiskers show a 1.5 interquartile range, outliers are marked with dots. C) PCA plot of seeds' transcriptomes (Seurat with sctransform normalization). D-F) PCA plots with the number of sequenced reads, identified genes, and the fraction of intergenic reads.

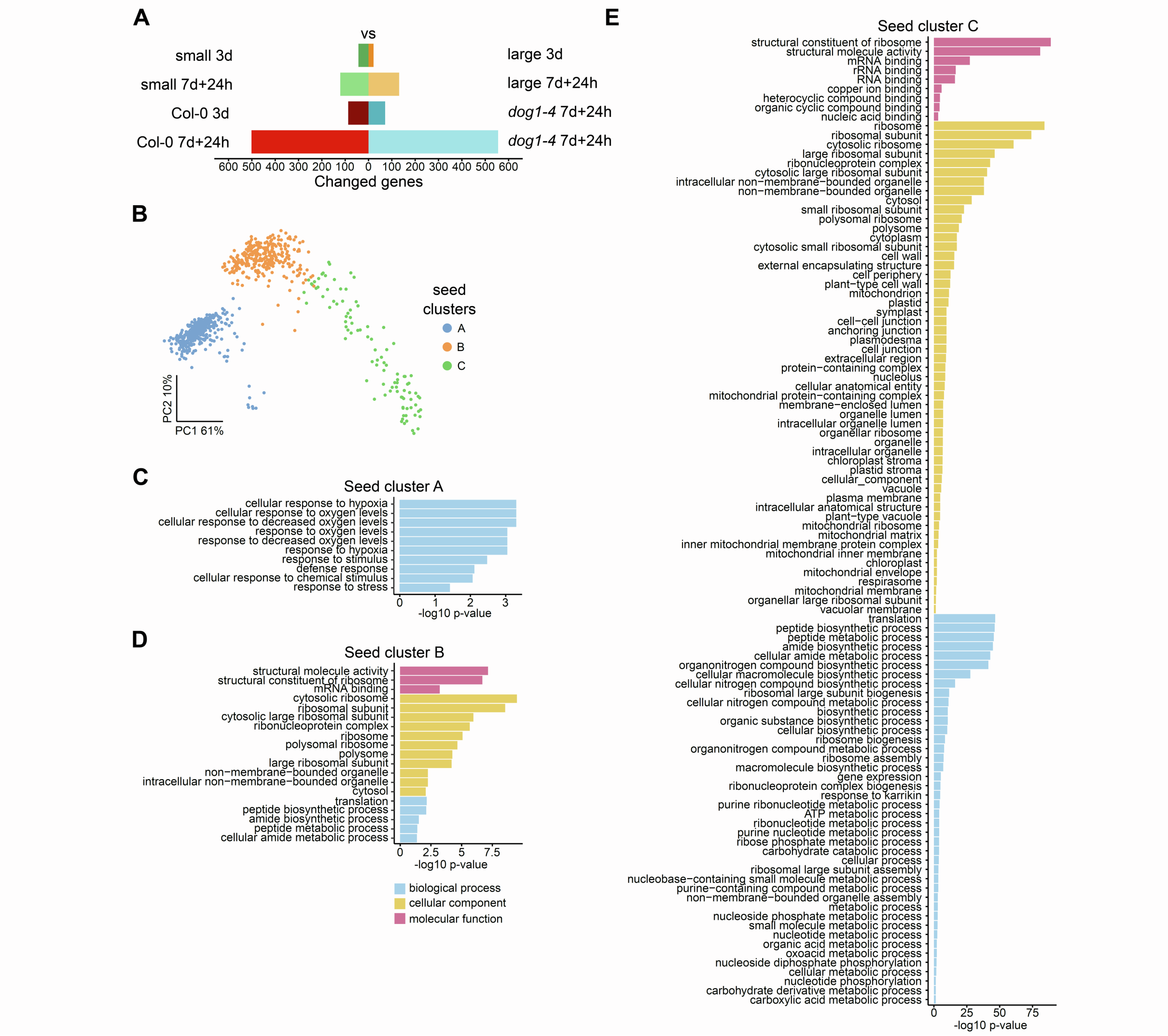

Supplemental Figure 5. The expression of translation-related genes is upregulated in the "tail" of the 7d+24h condition in the PCA plot. A) Number of genes identified as upregulated in seeds when different conditions were compared (Seurat FindMarkers Wilcoxon Rank Sum test; adjusted p-value < 0.05; $|\log_2 FC| > \log_2(1.2)$). B) PCA plot with three clusters of seeds identified by hierarchical clustering. C-E) GO terms enriched (gprofiler2) among genes whose expression is upregulated (Seurat FindMarkers Wilcoxon Rank Sum test; adjusted p-value $<$ 0.05; $|\log_2 FC| > \log_2(1.5)$) in each seeds' clusters. Colors show sources of gene ontologies.

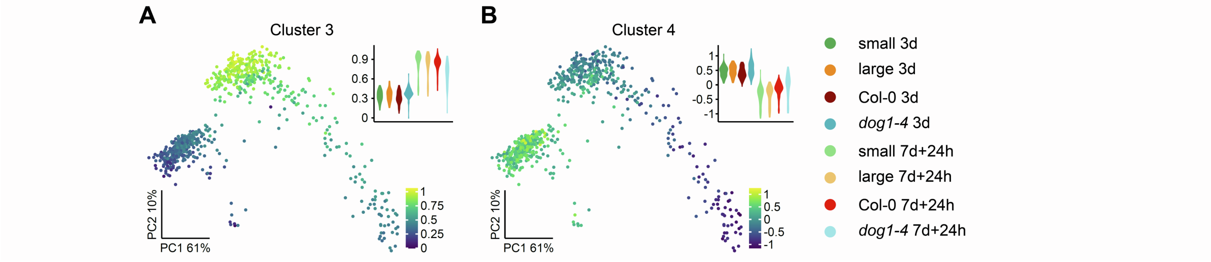

Supplemental Figure 6. Co-expressed gene groups. A-B) Gene expression signature values (mean normalized expression) of two other co-expressed gene groups overlaid on the PCA plot. Gene expression correlation among seeds was calculated (scran R package), and gene pairs with correlation > 0.5 were used for clustering (RBGL R package). Gene groups with at least 10 genes were kept. Identified groups were used to calculate signatures (Seurat AddModuleScore). Signature values spread for each condition are shown as a violin plot.

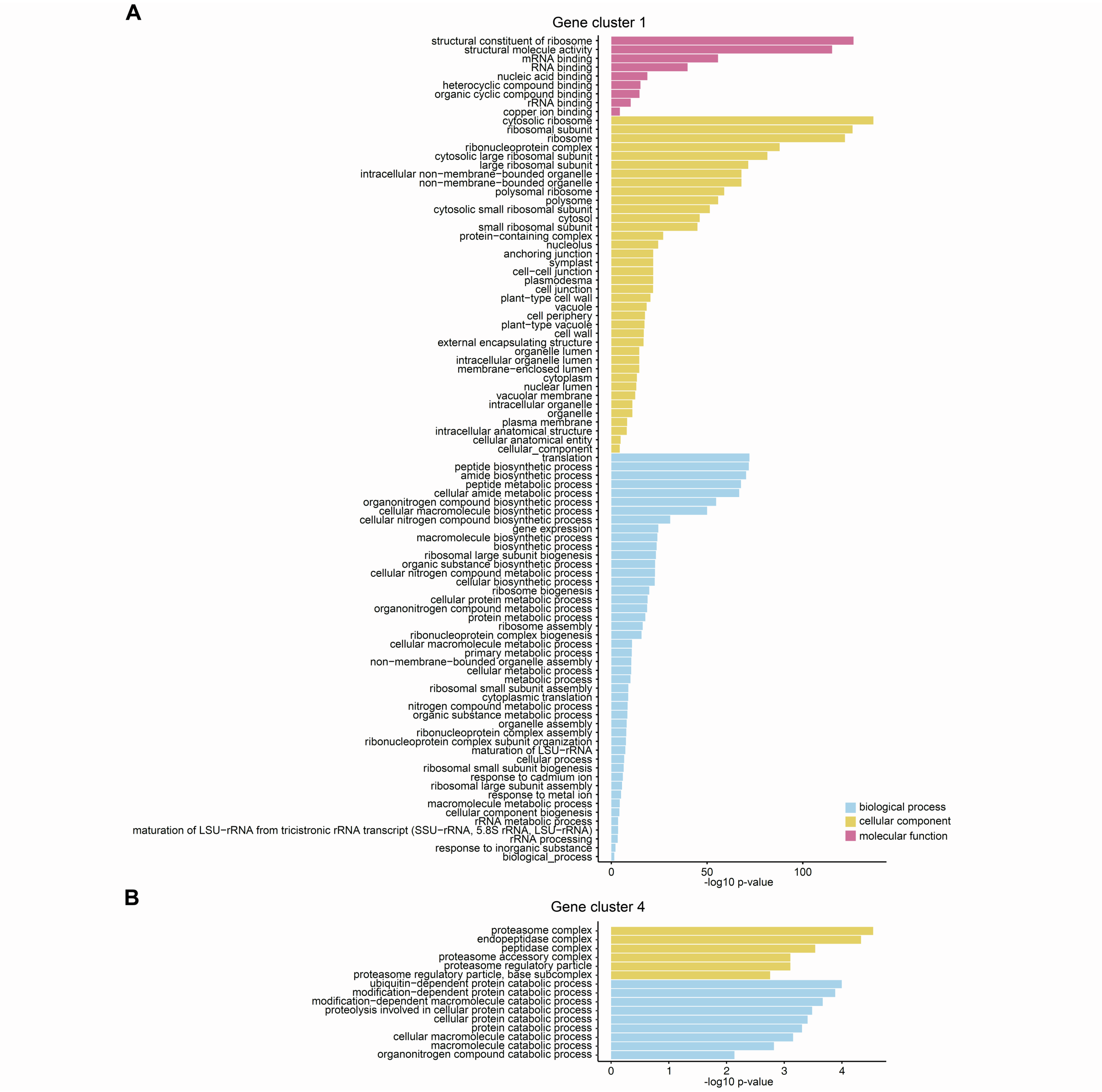

Supplemental Fig. S7. A-B) GO terms enriched (gprofiler2) among genes grouped in co-expressed cluster 1 (A; Figure 5A) and co-expressed cluster 4 (B; Supplemental Figure 6B). Colors show sources of gene ontologies.

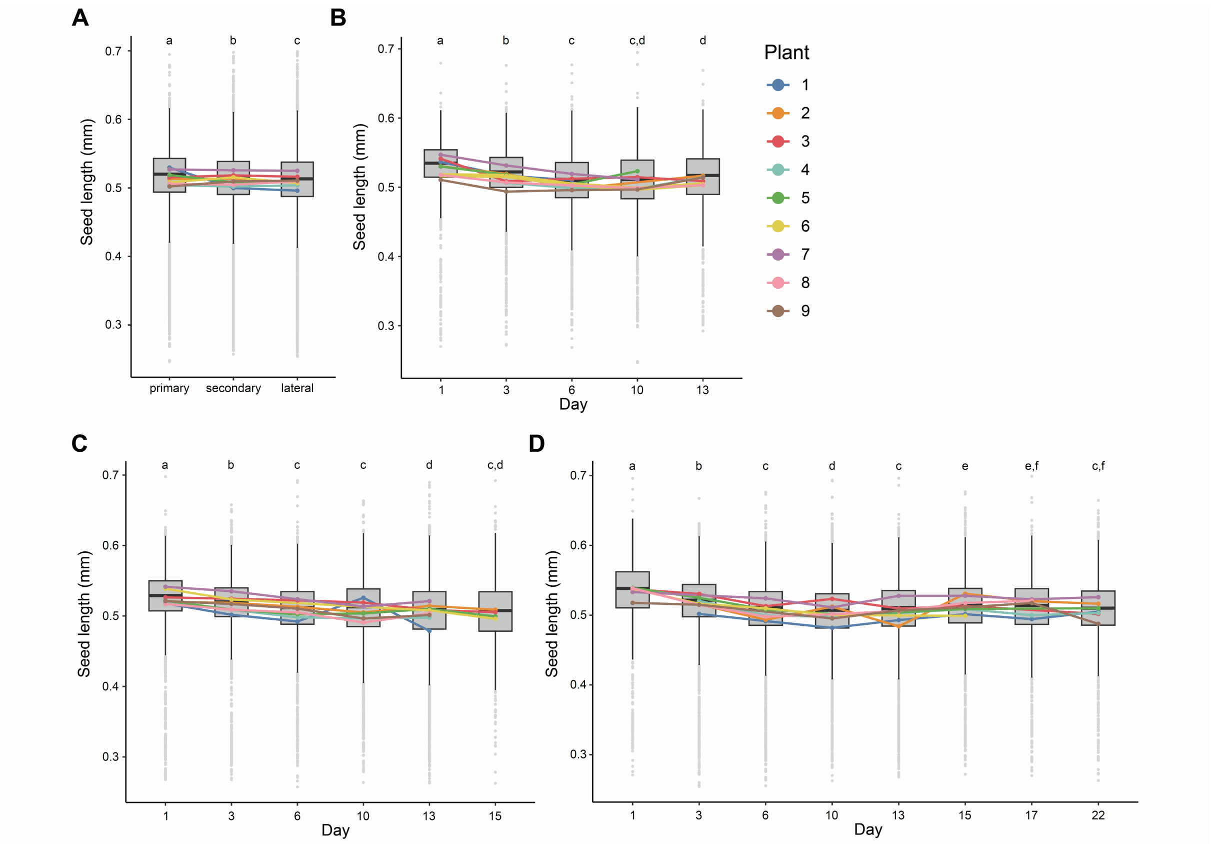

Supplemental Figure 8. Seed size slightly changes during the reproductive stage in Arabidopsis. A) Comparison of seed sizes collected from different parts of the plant (primary, secondary and lateral shoots). B) Comparison of seed sizes collected on different days from the primary shoot. C) Comparison of seed sizes collected on different days from secondary shoots. C) Comparison of seed sizes collected on different days from lateral shoots. Seeds were collected from nine plants. The letters above show the results of the pairwise Wilcoxon test (p-value < 0.001) performed for seeds pooled from all plants.
