# Supplementary material for: Dormancy heterogeneity among Arabidopsis thaliana seeds is linked to individual seed size
Source: Plant Commun. 2023 Oct 12;5(2):100732. doi: 10.1016/j.xplc.2023.100732 (PMC10873894; doi:10.1016/j.xplc.2023.100732)
Supplement: Document S2. Article plus supplemental information [file mmc6.pdf]

Plant Communications
Research article

CellPress
Partner Journal

Dormancy heterogeneity among *Arabidopsis thaliana* seeds is linked to individual seed size

Michal Krzyszton[1,5,*], Sebastian P. Sacharowski[1,5], Veena Halale Manjunath[1], Katarzyna Muter[1], Grzegorz Bokota[2], Ce Wang[1], Dariusz Plewczyński[2,3], Tereza Dobisova[4] and Szymon Swiezewski[1,*]

[1]Laboratory of Seeds Molecular Biology, Institute of Biochemistry and Biophysics, PAS, 02-106 Warsaw, Poland

[2]Laboratory of Functional and Structural Genomics, Centre of New Technologies, University of Warsaw, Warsaw, Poland

[3]Laboratory of Bioinformatics and Computational Genomics, Faculty of Mathematics and Information Science, Warsaw University of Technology, Warsaw, Poland

[4]Labdeers Ltd., 680-01 Boskovice, Czech Republic

[5]These authors contributed equally to this article.

***Correspondence: Szymon Swiezewski (sswiez@ibb.waw.pl), Michal Krzyszton (mkrzyszton@ibb.waw.pl)**

https://doi.org/10.1016/j.xplc.2023.100732

ABSTRACT

Production of morphologically and physiologically variable seeds is an important strategy that helps plants to survive in unpredictable natural conditions. However, the model plant *Arabidopsis thaliana* and most agronomically essential crops produce visually homogenous seeds. Using automated phenotype analysis, we observed that small seeds in Arabidopsis tend to have higher primary and secondary dormancy levels than large seeds. Transcriptomic analysis revealed distinct gene expression profiles between large and small seeds. Large seeds have higher expression of translation-related genes implicated in germination competence. By contrast, small seeds have elevated expression of many positive regulators of dormancy, including a key regulator of this process, the *DOG1* gene. Differences in *DOG1* expression are associated with differential production of its alternative cleavage and polyadenylation isoforms; in small seeds, the proximal poly(A) site is selected, resulting in a short mRNA isoform. Furthermore, single-seed RNA sequencing analysis demonstrated that large seeds resemble *DOG1* knockout mutant seeds. Finally, on the single-seed level, expression of genes affected by seed size is correlated with expression of genes that position seeds on the path toward germination. Our results demonstrate an unexpected link between seed size and dormancy phenotypes in a species that produces highly homogenous seed pools, suggesting that the correlation between seed morphology and physiology is more widespread than initially assumed.

Key words: seed size, dormancy, DOG1, *Arabidopsis*

Krzyszton M., Sacharowski S.P., Manjunath V.H., Muter K., Bokota G., Wang C., Plewczyński D., Dobisova T., and Swiezewski S. (2024). Dormancy heterogeneity among *Arabidopsis thaliana* seeds is linked to individual seed size. Plant Comm. **5**, 100732.

INTRODUCTION

Multiple plant species are characterized by heterocarpy or heterospermy, which is the production of morphologically and physiologically different types of fruits and seeds, respectively. The extent and significance of this phenomenon have been extensively analyzed and discussed in recent reviews (Matilla et al., 2005; Liu et al., 2018; Gianella et al., 2021). By contrast, most agronomically important plants, including wheat, corn, and rice, produce morphologically similar seeds. Likewise, the model plant *Arabidopsis thaliana* has visually very homogenous seeds that can nonetheless vary in physiological properties such as germination time under optimal and stress conditions (Abley et al., 2021; Batlla et al., 2022; Krzyszton et al., 2022). Seed morphological and physiological properties, including size, differ among Arabidopsis accessions (Herridge et al., 2011; Vidigal et al., 2016), enabling the identification of numerous loci affecting this feature (Krannitz et al., 1991; Herridge et al., 2011; Gnan et al., 2014; Ren et al., 2019). Moreover, both Arabidopsis seed shape and size are affected by mutations in multiple genes involved in diverse developmental and hormonal pathways (reviewed in Orozco-Arroyo et al., 2015; Li and Li, 2016). These include genes implicated in responses to

Published by the Plant Communications Shanghai Editorial Office in association with Cell Press, an imprint of Elsevier Inc., on behalf of CSPB and CEMPS, CAS.

This is an open access article under the CC BY license (http://creativecommons.org/licenses/by/4.0/).

hormones such as abscisic acid (ABA) (Xi et al., 2010), auxin (Liu et al., 2023), cytokinins (Riefler et al., 2005; Liu et al., 2020), brassinosteroids (Jiang et al., 2013), and ethylene (Robert et al., 2008), as well as genes that directly regulate seed development (Leon-Kloosterziel et al., 1994; Garcia et al., 2003; Robert et al., 2008; Zhou et al., 2009; Doughty et al., 2014; Cheng et al., 2018; Yu et al., 2023). Finally, Arabidopsis seed size is also affected by allocation of storage materials during seed maturation (Herridge et al., 2011; Li et al., 2022). All these previous works have demonstrated that multiple environmental and endogenous inputs influence seed size. However, combined analysis of Arabidopsis morphological and linked physiological seed variability within a single seed pool has rarely been performed (Elwell et al., 2011; Vidigal et al., 2016).

One of the physiological features shown to differentiate seeds is the level of dormancy, which is the ability to postpone germination despite favorable conditions (Nonogaki, 2019). Primary dormancy is established during seed maturation, and its variable levels allow for diversification of germination time in the seed population (Bradford, 2018). Even once initial dormancy is alleviated, imbibed seeds that encounter environmental stress may re-induce the dormant state. This secondary dormancy is crucial for the long-term survival of seeds in natural habitats (Buijs, 2020). Despite some differences, both primary and secondary dormancy depend on multiple regulatory pathways, including hormonal regulation by ABA (Buijs, 2020) and the *DELAY OF GERMINATION* (*DOG1*) gene (Bentsink et al., 2006; Buijs, 2020). Loss of *DOG1* gene expression leads to low primary and secondary seed dormancy (Bentsink et al., 2006; Buijs, 2020). Its expression is regulated on multiple levels (reviewed in Nonogaki, 2019), including the production of short and long *DOG1* transcript isoforms by alternative cleavage and polyadenylation (Cyrek et al., 2016). Both isoforms are tightly co-regulated in all tested conditions (Cyrek et al., 2016). Nevertheless, only the short isoform was shown to be functional, as it can complement the *DOG1* mutant phenotype (Cyrek et al., 2016).

Our recent work described gene expression variability in a genetically identical seed population during stress treatment (Krzyszton et al., 2022). This treatment resulted in secondary dormancy establishment in a fraction of the seeds, which was reflected by genes' expression variability that positions the seeds on the germination–dormancy axis (Krzyszton et al., 2022). However, it is unclear whether any identified transcriptomic differences correlate with seed morphological properties. Importantly, a link between low seed mass and high dormancy was established in ecological studies when multiple plant species were compared (Venable and Brown, 1988; Volis and Bohrer, 2013; Liu et al., 2017).

Here, we asked whether relatively small differences in seed size observed within a seed pool are linked to differences in seed physiology and transcriptomic profiles in Arabidopsis. Automated seed sowing coupled with size analysis enabled us to observe that small seeds tended to have higher primary and secondary dormancy levels. To determine the molecular sources of this behavior, we performed a 3′ RNA sequencing (3′ RNA-seq) analysis and discovered that large seeds had higher expression of translation-related genes previously shown to be associated with germination potential (Buijs et al., 2020). Our results revealed that ABA-responsive genes and the *DOG1* gene have higher expression in small seeds, accompanied by *DOG1* preferential proximal cleavage and polyadenylation site selection. Finally, we performed single-seed RNA-seq analysis during and after secondary dormancy induction and observed that large seeds tend to be more similar to the dormancy-deficient *dog1-4* mutant (Bentsink et al., 2006). Moreover, the single-seed analysis indicated a correlated continuum of gene expression differences among size- and germination-related genes.

RESULTS

Arabidopsis seed size is correlated with dormancy level

In our analysis of single-seed morphological characteristics, we used a recently developed Boxeed robot (Labdeers). We focused on seed size as the most basic seed morphological property (Supplemental Figure 1A). This seed feature showed close to a normal distribution in assayed seed pools, with some differences among biological replicates (n = 4) in the position of medians and skewness (Figure 1A; Supplemental Figure 1B). Such differences have been reported previously and may result from slight differences in growth conditions or morphology of the mother plant (Elwell et al., 2011; Herridge et al., 2011). We used freshly harvested Arabidopsis Col-0 seeds to assay primary dormancy levels (Supplemental Figure 1C) and observed that seed sizes of germinated and non-germinated seeds tended to differ in each assayed seed pool (Figure 1B). Importantly, non-germinated seeds tended to be smaller than germinated ones (Figure 1B). To further investigate the differences between small and large seeds, we used the secondary dormancy induction protocol. Uniformly germinating seeds that had already lost their primary dormancy (after-ripened) were incubated in darkness at 30°C and then transferred to permissive conditions (Ibarra et al., 2016; Krzyszton et al., 2022). This treatment resulted in inhibition of germination for 31%–72% of seeds (Supplemental Figure 1D). Notably, large seeds also showed higher germination levels in this experiment (Supplemental Figure 1E). A similar conclusion was reached when the dry seed area was assessed manually using a microscope and ImageJ software, followed by secondary dormancy induction (Schneider et al., 2012) (Supplemental Figure 1F and 1G).

Next, we assayed primary dormancy in three batches of single siliques. Siliques were collected from different plants at the same time to ensure a similar developmental stage. Germination was analyzed at a single-silique level and showed that 0% to 100% of the seeds were dormant. Importantly, we noticed that siliques with larger seeds showed a higher germination percentage than siliques with smaller seeds (Supplemental Figure 1H). In this experiment, we also analyzed germination time recorded on subsequent days after sowing. We observed that the largest seeds tended to germinate earliest, whereas smaller seeds germinated later or not at all (Figure 1C). This was consistent with stronger dormancy of smaller seeds, as it has been proposed that germination rate is closely related to dormancy level in Arabidopsis (Soppe and Bentsink, 2020).

Finally, to confirm our observations, we used the reverse approach. We first pre-selected 10% of the smallest and largest

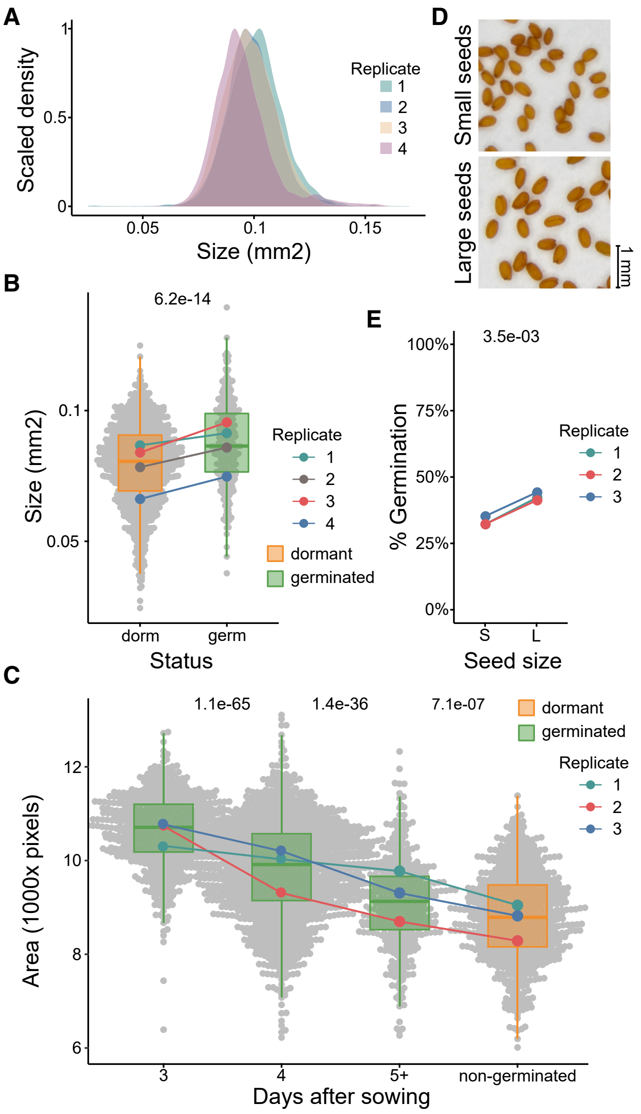

Figure 1. Small seeds are more dormant.
(A) Col-0 seed size distributions of four seed pools (*n* = 3802–5491).
(B) Size distribution of dry, freshly harvested Col-0 seeds (Supplemental Figure 1C). Mean values of seed sizes for dormant (dorm) and germinated (germ) seeds are shown as points for each of four replicates (*n* = 320–607). The *p value* of the Wilcoxon rank-sum test for comparison of seed sizes from all replicates is shown above the plot.
(C) Seed sizes and germination days of freshly harvested Col-0 seeds. The mean values of seed sizes are shown as points for each of the three replicates. The *p value* of the pairwise Wilcoxon rank-sum test (with false discovery rate correction) for the comparison of seed sizes from all replicates is shown above the plot.
(D and E) (D) The top 10% of the smallest and largest seeds were sorted, imaged, and tested **(E)** for germination after 7 days of secondary dormancy induction (*n* = 100). The *p value* of the two-sided paired *t*-test is shown above the plot. Seeds were sown and analyzed using a Boxeed robot. Seed germination was scored after 7 days.

seeds (Figure 1D; Supplemental Table 1) and then used these seed sub-pools for secondary dormancy induction. Again, small seeds turned out to be more dormant than large ones (Figure 1E). Importantly, an assessment of seed viability showed that small and large seeds had equally high chances of surviving the secondary dormancy induction treatment (Supplemental Figure 1I). Interestingly, using another pool of small and large seeds, we observed that the difference in germination percentage was also clear in the presence of ABA (Supplemental Figure 1J). This suggests that the physiological difference between small and large seeds is most likely ABA independent, as it persists despite an excess of exogenous ABA.

In summary, our results showed that smaller Arabidopsis seeds displayed higher dormancy levels when assayed for both primary dormancy and secondary dormancy induction. This suggests a close relationship between seed size and dormancy distributions among seeds.

Small and large Arabidopsis seeds differ in transcriptomic profiles

Next, we asked whether the observed link between seed size and dormancy level was reflected in the seed transcriptomes. Using the automated Boxeed system, we sorted seeds into small-, medium-, and large-size pools and performed 3′ RNA-seq. This method of transcriptomic analysis was selected because it is very time efficient and cost effective (Krzyszton et al., 2022). A principal-component analysis (PCA) plot of the 3′ RNA-seq results showed that the first principal component recapitulated seed size (Figure 2A), suggesting that size strongly influences the seed transcriptome. As expected, the largest number of differentially expressed genes was identified when extreme seed sizes were compared (248 and 611 up- and downregulated genes, respectively; false discovery rate <0.05) (Supplemental Figure 2A; Figure 2B; Supplemental Dataset 1). Only a few genes were unique to the comparison between the extremes and the medium-sized seeds (Supplemental Figure 2A). Interestingly, gene expression profiles showed a gradual decline or increase in gene expression when medium-size seeds were included (Figure 2B). Notably, Gene Ontology (GO) term analysis revealed that genes with higher expression in large seeds were enriched in many terms involved in translation, whereas genes with higher expression in small seeds were enriched in terms related to abiotic stress response, proteolysis, and seed storage materials (Supplemental Figure 2B and 2C). We noticed that genes differentially expressed between small and large seeds showed similarities to genes affected during secondary dormancy induction (Krzyszton et al., 2022). In that experiment, gene expression analysis among single seeds identified two co-expressed gene groups: one group with increased expression in seeds that were more advanced toward germination and a second group characteristic of seeds with higher dormancy levels. Based on these genes lists, we created a transcriptional germination competence index showing the position of a single seed on the supposed germination path (Krzyszton et al., 2022). Here, we showed that large seeds were characterized by high expression values of genes associated with germination competence, whereas small seeds showed high expression of genes linked to seed dormancy (Figure 2C). Finally, a closer examination of genes whose expression was affected by seed size (Supplemental Dataset 1) revealed enrichment of mRNAs related to seed storage materials and ABA-induced desiccation tolerance in small seeds. Also, several well-described regulators of seed biology were expressed in a size-dependent manner.

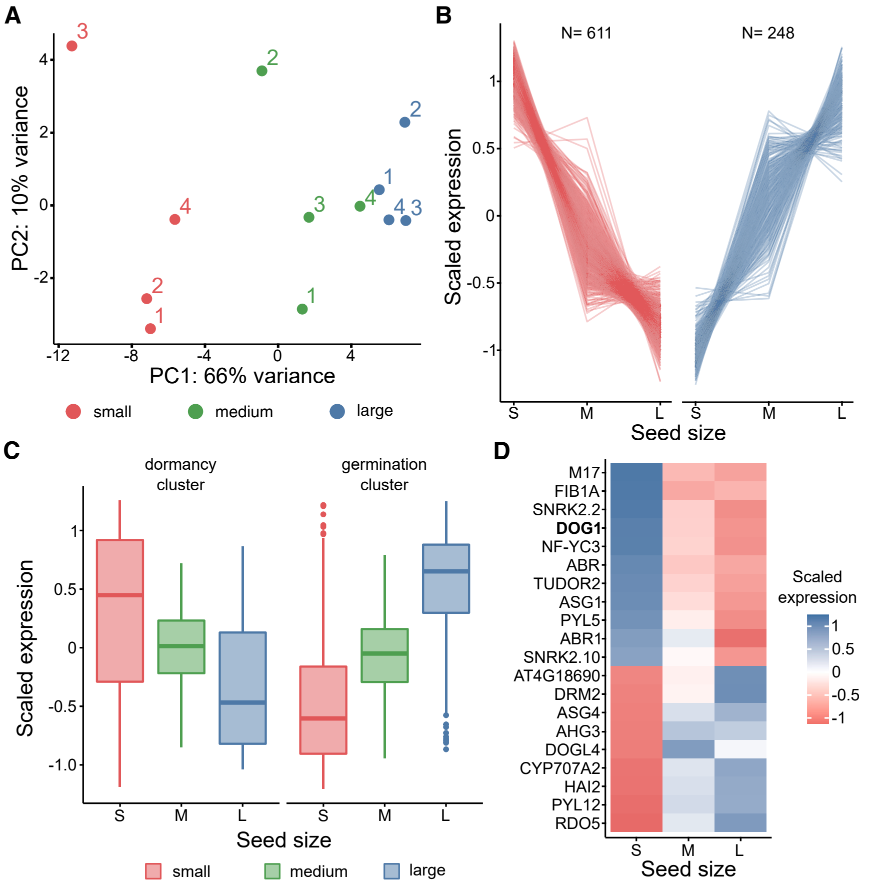

Figure 2. Small and large seeds differ in transcriptome profiles.

(A) PCA plot (DESeq2, rlogTransformation) for sequenced libraries showing the first two principal components (PCs). PC1 values are positively correlated with the size of the seeds used for library preparation. Numbers above the points denote biological replicates.

(B) Differentially expressed genes (DESeq2, adjusted p <0.05) between small and large seeds show a gradual change in expression through medium-size seeds. Expression was scaled before plotting; N, number of affected genes.

(C) Seed size is linked to the expression of dormancy- and germination-related gene groups (Krzyszton et al., 2022). The level of normalized gene expression among small, medium, and large seeds is shown using boxplots. Boxplot whiskers show a 1.5 interquartile range; outliers are marked with dots.

(D) Selected seed biology-related genes show differences in expression between seeds of different sizes. The heatmap shows the average normalized and scaled expression of four biological replicates.

These included genes with higher expression in large seeds: the *CYP707A2* ABA degradation enzyme (Kushiro et al., 2004), *AHG3* and *HAI2* phosphatases that negatively regulate the ABA response (Yoshida et al., 2006; Bhaskara et al., 2012), the *PYL12* ABA receptor (Zhao et al., 2020), and *RDO5*, a dormancy regulator (Xiang et al., 2014). At the same time, small seeds were characterized by higher expression of the *PYL5* ABA receptor (Zhao et al., 2020), the *SnRK2.2* and *SnRK2.10* kinases, which are positive regulators of the ABA response (Fujii et al., 2011), and, most notably, *DOG1*, a key seed dormancy regulator in Arabidopsis (Footitt et al., 2020; Batlla et al., 2022) (Figure 2D). Many of these genes are involved in ABA-regulated functions, consistent with the reported role of ABA in seed germination and dormancy control (Nonogaki, 2019; Buijs, 2020). We confirmed the differences in expression of selected genes between small and large seeds using RT–qPCR and found that seven out of eight showed changes consistent with those observed in the 3′ RNA-seq data (Supplemental Figure 2D).

DOG1 expression is correlated with seed size

Among the genes with altered expression, *DOG1* is tightly and quantitatively linked with dormancy changes. The *DOG1* gene is a well-known quantitative trait locus (QTL) and genome-wide association study candidate for seed dormancy (Bentsink et al., 2006; Kerdaffrec et al., 2016). *DOG1* expression is correlated with dormancy among *A. thaliana* accessions (Chiang et al., 2011), when seeds are matured in different temperatures (Kendall et al., 2011), and during seed dormancy cycling in soil (Footitt et al., 2020). In agreement with these findings, *dog1* mutant heterozygous seeds show a level of dormancy intermediate between that of the wild type and the homozygous mutant (incomplete dominance) (Fedak et al., 2016). As a result, *DOG1* gene expression is tightly linked with dormancy, such that even minor changes in its expression are reflected in the phenotype (Bentsink et al., 2006; Kerdaffrec et al., 2016). Although this has not been explored extensively for other genes involved in dormancy control whose expression was affected in our RNA-seq experiment, none of them have been reported to show such a strong expression correlation with dormancy level. For these reasons, we focused here on exploring the cause of *DOG1* differential expression and its role in establishing dormancy differences between seeds of various sizes.

Alternative cleavage and polyadenylation of *DOG1* pre-mRNA results in the production of long and short mRNA isoforms that have been shown to be co-regulated during development and in response to an external environmental stimulus (Cyrek et al., 2016). Here, examination of 3′ RNA-seq profiles revealed that low levels of *DOG1* mRNA in large seeds were associated with decreased expression of the short isoform, whereas levels of the long isoform were unchanged or slightly increased (Figure 3A) (Cyrek et al., 2016). Consequently, the ratio of isoform levels changed with seed size (Figure 3B). Using isoform-specific RT–qPCR analysis, we confirmed that small seeds showed high levels of proximally polyadenylated *shDOG1* mRNA, whereas long *DOG1*, which results from distal poly(A) site selection, was unaffected (Figure 3C). The fact that small seeds were more dormant and showed higher expression of the short,

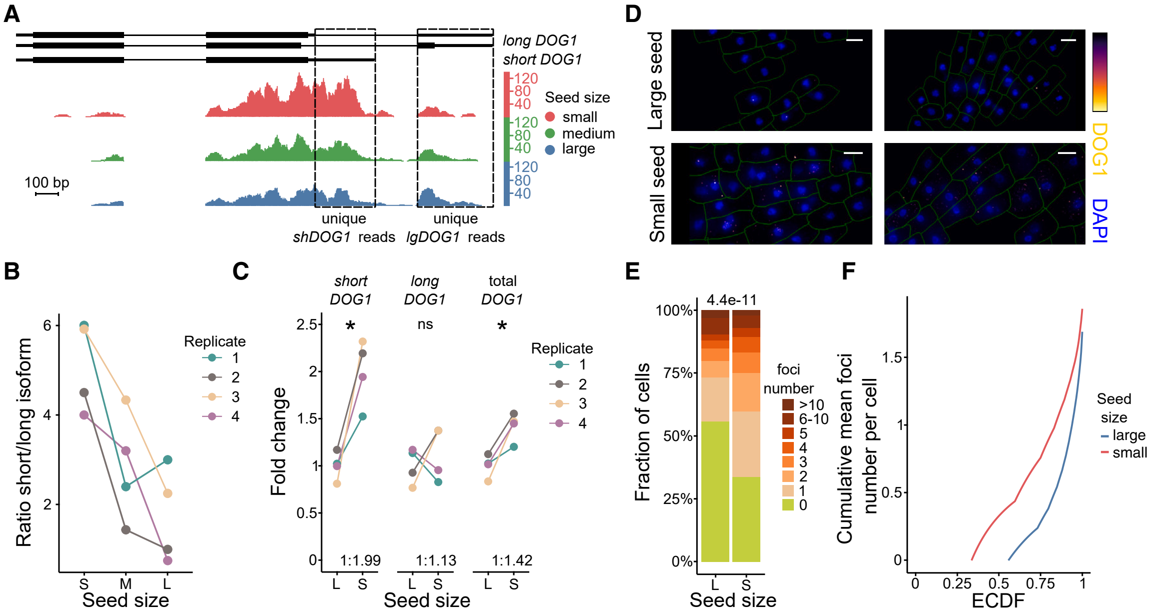

Figure 3. *DOG1* expression differentiates seeds of different sizes.

(A) 3′ RNA-seq reads at the *DOG1* locus. Replicates of libraries for seeds of different sizes were combined, normalized, and plotted using the Integrated Genome Browser. Selected *DOG1* isoforms are shown, with boxes marking regions used to estimate expression of isoforms.

(B) Ratio of short and long *DOG1* mRNA isoforms differentiates small and large seeds. The ratio of read counts for two regions from **(A)**. Each of the four biological replicates is shown (S, small; M, medium; L, large).

(C) RT–qPCR analysis of total *DOG1* transcript levels and those of its main isoforms in four replicates of large and small Col-0 seeds. Fold change relative to the average value for large seeds is shown for each transcript. * $p < 0.05$; ns, non-significant (paired *t*-test). Ratios of isoform levels are shown above the x axis. *UBC21* mRNA was used as a reference.

(D) z stack max-projection images of smFISH for *DOG1* RNA. The “Inferno” color scale is used for the intensity of fluorescence from the Quasar670 fluorophore (*DOG1*). The blue color shows DAPI fluorescence (nuclear staining). Scale bar, 20 μm.

(E) Bar plot showing the number of *DOG1* transcripts per cell based on the smFISH experiment. Pearson’s chi-squared test *p value* is shown above the plot.

(F) Plot of the empirical cumulative distribution function of cumulative mean foci number.

proximally polyadenylated *DOG1* isoform agrees with the notion that *shDOG1* is responsible for production of the functional DOG1 protein (Cyrek et al., 2016). The long *DOG1* mRNA isoform has been shown to be alternatively spliced, and this splicing is controlled by Pol II processivity (Dolata et al., 2015; Cyrek et al., 2016). However, we did not observe strong differences in long *DOG1* mRNA splicing isoform ratios between small and large seeds (Supplemental Figure 3A).

We also performed a single-molecule fluorescence *in situ hybridization* (FISH) (smFISH) analysis of *DOG1* transcripts in embryonic root tips (Montez et al., 2023) from small and large seeds (Figure 3D; Supplemental Figure 3B). This enabled us to observe that small seeds were characterized by a larger fraction of cells with detectable *DOG1* transcripts compared with large seeds (Figure 3E). Interestingly, a few outlier cells from root tips of large seeds had many *DOG1* transcripts, which made the mean number of *DOG1* transcripts per cell comparable between small and large seeds (Figure 3F).

Our analysis of dormancy-deficient *dog1-4* knockout mutant seeds did not reveal any significant changes in absolute seed size or seed size distribution compared with Col-0 (Supplemental Figure 3C). In agreement with published work (Krzyszton et al., 2022), the low dormancy of *dog1-4* mutant seeds was manifested in higher germination rates after secondary dormancy induction compared with wild-type seeds (Supplemental Figure 3D). Despite the overall low dormancy level of the *dog1-4* seeds, the remaining *dog1-4* seeds that did enter dormancy showed a skew toward small size (Supplemental Figure 3E). This difference was much less significant than that for wild-type seeds, presumably owing to the inability of most *dog1* mutant seeds to enter dormancy (Supplemental Figure 3D).

To conclude, our transcriptomic data showed that *DOG1* and other dormancy-related factors were differentially expressed across the seed population in a seed-size-dependent manner (Figure 2D), which suggested that seed size and dormancy levels are linked in Arabidopsis seeds. Only partial loss of dormancy in both large and *dog1* mutant seeds (Supplemental Figure 3D) agrees with previous works that revealed DOG1-independent contributors to dormancy control (Nakabayashi et al., 2012; Footitt et al., 2017, 2020). Moreover, the phenotype of *dog1-4* mutant seeds (Supplemental Figure 3C) showed that changes in dormancy may not influence seed size. This could reflect the fact that dormancy appears during the late stages of seed maturation after seed size has already been established. Importantly, dormancy levels can be modified by seed exposure to the environment (for example, during secondary

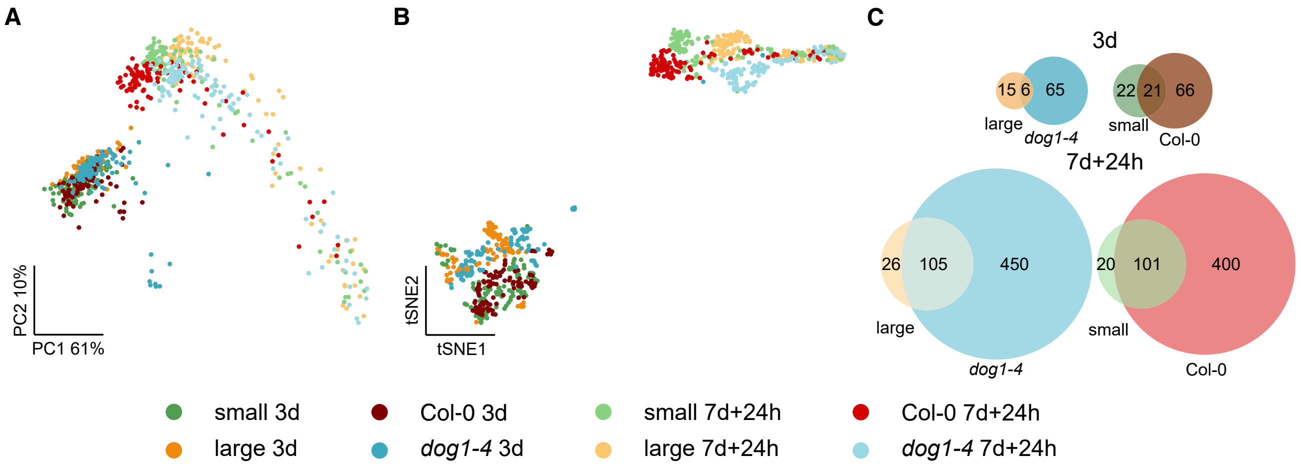

Figure 4. Single-seed RNA-seq reveals similarities between large seeds and *dog1-4* mutant seeds.
(A) PCA plot (Seurat with sctransform normalization) of seed transcriptomes shows that seeds are grouped by condition. Seeds from the 7d + 24h condition are grouped according to seed size/genotype, with a mixture of seeds in the tail.
(B) t-distributed stochastic neighborhood embedding plot (Seurat) of seed transcriptomes revealed the grouping of small/Col-0 and large/*dog1-4* mutants seeds for both 3d and 7d + 24h time points.
(C) Overlaps of affected genes in differential expression analysis (Seurat FindMarkers Wilcoxon rank-sum test; adjusted $p < 0.05$; $|\log_2 FC| > \log_2(1.2)$). Comparisons confirm similarities between small and Col-0 seeds and between large and *dog1-4* mutant seeds for both 3d and 7d + 24h time points. Numbers in Venn diagrams show the number of genes in each intersection.

dormancy establishment), whereas seed size is a stable value and that may blur even strong initial correlations.

Large seeds resemble *dog1* mutant seeds

To confirm the contribution of *DOG1* to seed size-related transcriptomes, we used our recently developed single-seed RNA-seq method (Krzyszton et al., 2022). In this approach, multiple single seeds originating from the same pool are subjected to low-coverage RNA-seq. Given the relatively narrow distribution of seed sizes in a seed pool, we decided to sequence 96 preselected small and large seeds from the Col-0 plant, which allowed us to avoid sequencing only the most common medium-sized seeds and thus increased the stringency of our analysis. This experiment was performed in parallel with the single-seed RNA-seq of Col-0 and *dog1-4* seeds during secondary dormancy induction published previously (Krzyszton et al., 2022). Both small/large and Col-0/*dog1-4* seeds were treated together and harvested on the third day (3d) of secondary dormancy induction and after 24 h of recovery following 7 days of induction (7d + 24h). Single-seed library 3′ RNA-seq preparation, sequencing, and data analysis were also performed together for both experiments. Quality controls of small and large seed libraries (Supplemental Figure 4A) showed a relatively low number of intergenic reads, pointing to the high quality of the library preparation (Supplemental Figure 4B). Positions of seeds on the PCA plot (Supplemental Figure 4C) were not determined by the intergenic read content, total number of sequenced reads, or number of identified genes (Supplemental Figure 4D–4F). Similar conclusions were also reached for Col-0 and *dog1-4* libraries (Krzyszton et al., 2022), validating the experimental approach. However, the expression of *DOG1* in imbibed seeds (Bentsink et al., 2006) was below the detection threshold of the single-seed RNA-seq method, and *DOG1* expression could not be assayed directly (Krzyszton et al., 2022).

The PCA plot showed that the seeds were grouped according to treatment. Interestingly, the grouping of seeds from the 7d + 24h time point showed a comet-like shape, with a long tail of seeds protruding from the main seed group (Figure 4A). To facilitate analysis of single-seed grouping, we prepared t-distributed stochastic neighborhood embedding (tSNE) visualization, which showed close associations of Col-0 with small seeds and *dog1-4* with large seeds at both time points (Figure 4B). To verify these pairwise similarities, we performed differential gene expression analysis between Col-0 and the *dog1* mutant as well as small and large seeds (Supplemental Figure 5A; Supplemental Dataset 2). Our observations revealed that the extent of affected genes in both conditions (3d and 7d + 24h) was very similar for both Col-0 and small seeds and *dog1-4* and large seeds (Figure 4C). These results supported the observed phenotypic similarity between large wild-type seeds and *dog1* mutant seeds and suggest that low expression of *DOG1* in large seeds is an important factor in shaping their transcriptome.

Size- and germination-related genes show similar expression patterns among single seeds

Hierarchical clustering of seeds revealed three major groups: one consisting of 3d-treated seeds, one containing the 7d + 24h-treated seeds that formed the "head of the comet", and a separate group with seeds from the "tail of the comet" (Supplemental Figure 5B). Differential gene expression analysis between identified seed groups showed that translation- and ribosome-related genes were strongly expressed in the protruding tail. This observation suggests active metabolism and conceivably represents the gradual advancement toward germination of these seeds compared with those in the second seed cluster of the 7d + 24h time point (Supplemental Figure 5C–5E; Supplemental Dataset 2). A similar pattern of co-expression of germination-related genes was observed by us previously and was negatively correlated with the expression of dormancy-associated genes

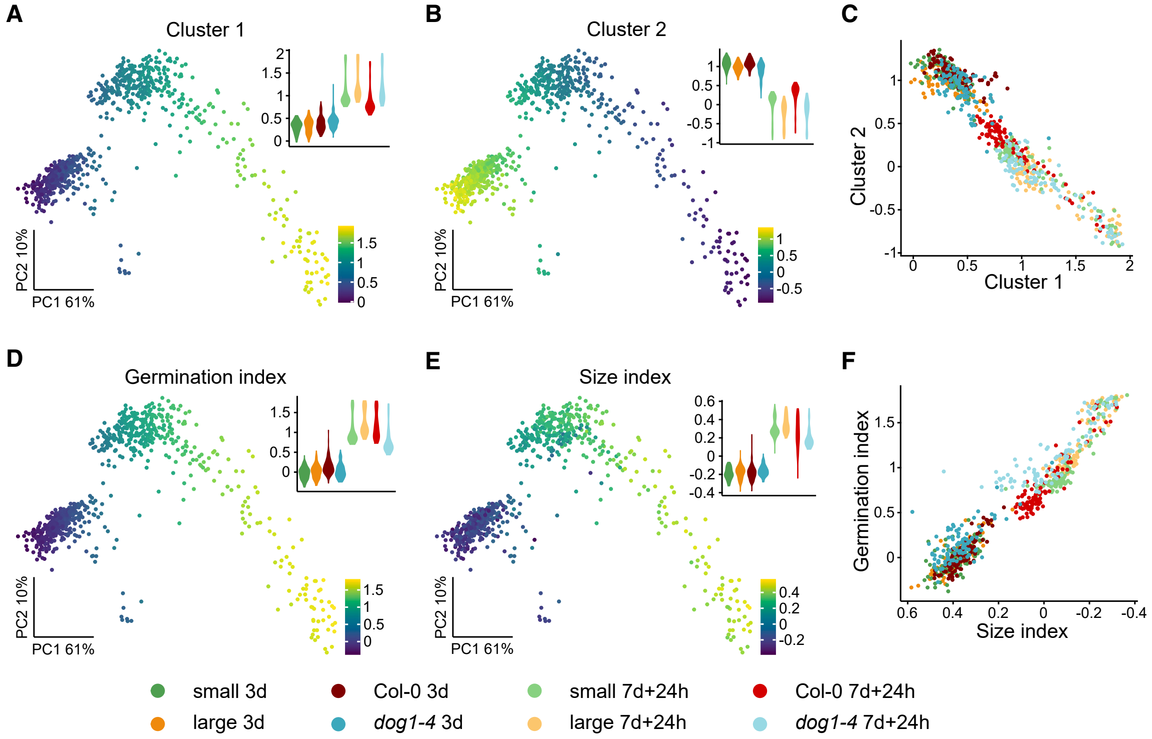

Figure 5. Genes affected by germination and seed size show a similar expression profile.

(A and B) Gene expression signature values (mean normalized expression) of two main co-expressed gene groups overlaid on the PCA plot. Gene expression correlation among seeds was calculated (scran R package), and gene pairs with correlation >0.5 were used for clustering (RBGL R package). Identified groups were used to calculate signatures (Seurat AddModuleScore). The spread of signature values for each condition is shown as a violin plot.

(C) Plot of group 1 and group 2 gene expression signature values for each seed.

(D) Germination competence signature overlaid on PCA plot. Composite signature values were calculated using the Vision R package on the basis of the expression of cluster 1 and cluster 2 genes.

(E) Seed size signature overlaid on PCA plot. Composite signature values were calculated using the Vision R package on the basis of differentially expressed genes between small and large seeds (mean normalized expression of genes with upregulated and downregulated expression had negative and positive input, respectively). The spread of signature values for each condition is shown as a violin plot.

(F) Size and germination signatures are correlated across the seeds.

(Krzyszton et al., 2022) (see also Figure 2C). On the basis of these gene groups and their negative expression correlation, we constructed a transcriptomic germination competence index that recapitulates the observed dormancy-to-germination axis (Krzyszton et al., 2022).

Here, the identification of co-expressed genes pointed to four gene clusters (Figure 5A and 5B; Supplemental Figure 6A and 6B; Supplemental Dataset 2). GO-term enrichment analysis showed that translation-related genes were the main component of cluster 1 (Supplemental Figure 7A). Consequently, cluster 1 genes showed the highest values of expression for the tail on the PCA plot (Supplemental Figure 6A). Clusters 2 and 3 showed no significant GO-term enrichment, and cluster 4 showed enrichment of genes involved in proteolytic function (Supplemental Figure 7B), which we also observed for small dry seeds (Supplemental Figure 2B). As before (Krzyszton et al., 2022), we observed a negative correlation between gene expression levels for the two main co-expressed gene groups (Figure 5C). Using these two gene groups, we created a transcriptional germination index as described previously (Krzyszton et al., 2022) that aligned seeds on the predicted path toward germination (Figure 5D).

Using genes that were differentially expressed between small and large dry seeds (Figure 2B), we created an index reflecting gene expression changes associated with seed size (Figure 5E). This new index illustrated transcriptional similarity to large seeds. Importantly, when we applied this index to our single-seed RNA-seq data, we found that it was positively correlated with transcriptional germination competence (Figure 5F). Both germination and size index values varied between seeds, even in the same condition, supporting the view that the underlying seed heterogeneity can be assayed only using a single-seed approach. In summary, single-seed RNA-seq revealed a gene expression gradient underlying transcriptional germination competence that was correlated with the expression of genes affected by seed size.

DISCUSSION

Our data showed a difference in dormancy levels between small and large Arabidopsis seeds. However, it is impossible to predict the exact seed germination percentage based on the mean seed size of the seed pool, as well as whether a specific seed will germinate based on its size. This is because a seed of a specific size in one pool may be described as large, whereas in another

pool it may be positioned in the middle of the size distribution. As a result, we conclude that in a seed pool collected from a single plant, smaller seeds have a higher chance of being dormant than large ones. This suggests that the probability of germination is linked to the relative position in a seed size distribution rather than to the absolute seed size (see Figure 1A). This dependence on the seed distribution resembles population-based threshold models that have been proposed to explain non-uniform seed germination (Bradford, 2018). Based on the fact that seed sizes in the tested pools differed, we hypothesized that germination percentages in the pools would be dependent on mean seed size. Although this tendency was true for single siliques (Supplemental Figure 1H), we did not observe it so clearly for larger pools (Supplemental Figure 1C).

Seed size and dormancy levels have been shown to reflect seed maturation conditions on the mother plant (Herridge et al., 2011; Burghardt et al., 2016). The observed link between dormancy and seed size could suggest that both traits independently mirror seed maturation conditions. This may explain the lack of a strong causative connection between seed size and dormancy. Our observations show that seed size fluctuates to some extent during plant development. We measured seed sizes on primary, secondary, and lateral shoots throughout the span of Arabidopsis reproduction. We did not observe major differences in seed size originating from different shoot types (Supplemental Figure 8A), but seed size decreased slightly with time on all three shoot parts (Supplemental Figure 8B–8D). Nonetheless, the variance in seed size within each pool and between plants was much larger than that between developmental time points. Based on this observation, we anticipate that dormancy and transcriptomic differences between small and large seeds will be strongly affected by the growth conditions of the mother plant.

The relationship between seed physiology and morphology has not been extensively studied in Arabidopsis. One study showed that smaller seeds germinate more slowly than large ones when seeds are divided using sieves (Elwell et al., 2011). Also, the production of small seeds is associated with stronger dormancy among different ecotypes (Vidigal et al., 2016). However, other work did not show any strong correlations between seed size and germination under multiple conditions when a set of recombinant inbred lines was assayed (Joosen et al., 2012). Our work showed a negative relationship between seed size and dormancy levels in *A. thaliana*. This has been observed previously for only a few heterospermic species (Larios et al., 2014; Volis, 2016; Xu et al., 2017; Nemati et al., 2022). Importantly, recent work showed that dimorphic seeds from single fruit of *Xanthium strumarium* differed in *DOG1* gene expression. Consistent with our work, smaller, more dormant seeds of this species were also characterized by high *DOG1* transcript levels (Nemati et al., 2022). Moreover, it has been shown that legume species with small seeds growing in temperate climates have a higher chance of using dormancy as an ecological strategy (Rubio De Casas et al., 2017). However, a large comparison among hundreds of phylogenetically diverse species showed no or only a weak negative association between seed size and dormancy (Jurado and Flores, 2005). Moreover, there have been several reports of species with higher dormancy linked to larger seeds (Gulden et al., 2004; Mira et al., 2019). A comprehensive literature analysis revealed that seed size was related to germination in most of the assayed 91 species: 58 species showed higher germination of large seeds, and only 11 showed higher germination of small seeds (Baskin and Baskin, 2014). Nonetheless, it is not clear whether the reported differences in germination were related to seed dormancy. We assume that a relationship between seed size and dormancy is strongly affected by species ecology (including dispersal strategy) and environment.

Initially, for the physiological tests, we used seed pools that were non-invasively, automatically analyzed for morphology and sown, allowing us to correlate seed size and dormancy (Figure 1B). On the basis of this result, we pre-selected small and large seeds for phenotypic, pooled 3′ RNA-seq, and single-seed RNA-seq analysis. We decided on this approach because we observed that the relationship of seed size to seed physiology was relatively weak, especially for the center of the seed size distribution. We needed to analyze nearly 1000 seeds to observe the relationship between seed size and dormancy level (Figure 1). Moreover, the single-seed RNA-seq method is relatively noisy and less sensitive than standard RNA-seq (Krzyszton et al., 2022). For this reason, we did not directly analyze single-seed transcriptomes in relation to seed size. However, the use of pools from the extremes of the distribution enabled us to find transcriptomic patterns hidden within the phenotypic variability (Figure 2) that could then be corroborated by single-seed analysis of pre-selected small and large seeds (Figure 4).

Transcriptomic analysis revealed that differences in dormancy levels between small and large seeds were reflected in gene expression. Surprisingly, among a few hundred genes known to affect seed size, only a few showed differential expression in our experiments. This may be explained by the fact that genes that affect seed size are mostly expressed during seed development or maturation, and their transcripts have already been removed before the dry stage. Alternatively, it suggests that size variability within seed lots is controlled by additional, unknown mechanisms. Translation-related genes were highly expressed in large seeds, suggesting that such seeds are ready for germination. Concordantly, recent reports showed that expression levels of translation-related genes are an important hallmark of a decrease in seed dormancy (Buijs et al., 2020; Krzyszton et al., 2022). Genes associated with desiccation tolerance and abiotic stress response were enriched in small seeds, indicating their higher dormancy levels. We also observed different levels of ABA response regulators in small and large seeds, consistent with the well-known role of ABA as a negative regulator of seed germination (Nonogaki, 2019). Despite that, we observed similar responsiveness of small and large seeds to ABA (Supplemental Figure 1J). The observed seed-size-dependent differences in gene expression resembled the transcriptomic germination competence index that aligned seeds on the path toward germination (Krzyszton et al., 2022). We confirmed this using a single-seed RNA-seq approach, which demonstrated transcriptomic heterogeneity and a strong relationship between germination- and size-related genes. This suggests that genes whose expression level is affected by seed size have a critical role in the regulation of seed biology.

Small seeds showed higher expression of the *DOG1* gene. Notably, single-seed RNA-seq revealed that large seeds demonstrated transcriptomic similarities to *dog1* mutant seeds (Figure 4C), suggesting that *DOG1* downregulation contributed to the large seeds' gene expression profile. Moreover, we observed a higher number of cells expressing *DOG1* transcripts in the root tips of small seeds than of large seeds. These results were consistent with a reduced overall dormancy of *dog1* mutant and large seeds. *DOG1* is a key player in dormancy control, and its expression is extensively regulated (Cyrek et al., 2016; Fedak et al., 2016; Nonogaki, 2019). *DOG1* expression is subject to alternative cleavage and polyadenylation control, among other mechanisms. Use of the proximal cleavage and polyadenylation site results in production of a short *DOG1* isoform that is sufficient for dormancy establishment (Cyrek et al., 2016). To date, neither conditions nor tissue specificity have been reported to control the selection of *DOG1* alternative cleavage and polyadenylation sites. Here we showed that, in addition to high *DOG1* expression, small seeds showed preferential selection of the proximally terminated *DOG1* isoform. This finding supports the conclusion that dormancy distribution among Arabidopsis progeny is affected by seed size and that transcriptional regulation of *DOG1* cleavage and polyadenylation site choice contributes to this mechanism. However, the observed smaller size of non-germinated *dog1* mutant seeds suggests a role for other players in linking seed size and dormancy levels.

We observed downregulation of *DOG1* and upregulation of *RDO5* mRNAs in large seeds that showed low dormancy. However, the change in *RDO5* expression was not confirmed by RT–qPCR. In contrast to the *DOG1* gene, no correlation between *RDO5* gene expression and dormancy has been reported among accessions (Kerdaffrec et al., 2016), although both DOG1 and RDO5 are positive regulators of dormancy (Xiang et al., 2014). Therefore, upregulation of *RDO5* in large, low-dormancy seeds can be explained by the fact that, apart from in *rdo5* mutants (Xiang et al., 2014), *RDO5* expression has not been reported to be correlated with dormancy strength. In addition, genetic analysis of the *rdo5 dog1* double mutant indicated a lack of additivity and a stronger effect of the *DOG1* gene (Xiang et al., 2014).

Counterintuitively, our results showed that the transcriptome of small seeds was enriched in mRNAs encoding proteins involved in the accumulation of seed storage materials (Supplemental Dataset 1). Recent work suggested a role for DOGL4 protein in the induction of seed storage- and late embryogenesis-related mRNAs (Sall et al., 2019). However, our transcriptomic results showed that *DOGL4* was downregulated in small seeds (Figure 2D), and the *dogl4* mutant has been reported to show no changes in seed dormancy (Sall et al., 2019). A double mutant of the *CYP707A1* and *CYP707A2* ABA catabolism genes also had a higher accumulation of seed storage and desiccation-related proteins (Chauffour et al., 2019). This suggests that high ABA levels during late seed maturation stages positively regulate the allocation of seed storage materials (Chauffour et al., 2019). Importantly, ABA levels may differ substantially among individual seeds, even from the same silique (Kanno et al., 2010), and ABA is well known to inhibit germination largely independently of the DOG1 protein (Nakabayashi et al., 2012). This is in agreement with our results demonstrating the differential expression of factors involved in ABA response between small and large seeds. Interestingly, *CYP707A2* expression was upregulated in the transcriptomes of large seeds (Figure 2D). This observation corresponds to the upregulation of *CYP707A2* mRNA in the *dog1* mutant (Nakabayashi et al., 2012). A complicated interplay between ABA and DOG1 protein in seed biology and gene expression regulation has been reported (Bentsink et al., 2006; Nakabayashi et al., 2012). DOG1 protein interferes with components of the ABA signal transduction pathway (Nishimura et al., 2007; Née et al., 2017). On the other hand, *DOG1* gene expression is controlled by ABA (Yatusevich et al., 2017; Chauffour et al., 2019) and requires the ABA pathway to control primary dormancy (Bentsink et al., 2006; Dekkers et al., 2016). Consistent with these findings, the ABA and DOG1 pathways have been shown to control dormancy partially independently (Bentsink et al., 2006; Graeber et al., 2014). Therefore, we postulate that ABA and DOG1 protein can act in a complicated, partially parallel manner to contribute to the final control of the link between seed size and dormancy.

Our work demonstrates that seed pools that seem to be highly homogenous may exhibit important morphological differences that affect the basis of seed physiology. We show that this hidden heterogeneity among Arabidopsis seeds is manifested in variable dormancy levels that are linked to seed size and are underpinned by transcriptional diversity among seeds.

METHODS

Plant material and germination assays

Col-0 and the *dog1-4* mutant (Bentsink et al., 2006) were used. To obtain seeds for experiments, plants were grown in a greenhouse under a long-day photoperiod at 22°C. For secondary dormancy induction, dry stored seed pools (stored for at least 6 months) were tested for residual primary dormancy, and seeds were then sown on plates with water-soaked blue paper. Plates were sealed with Parafilm and incubated in the dark at 30°C for 3 or 7 days. Seed recovery and germination tests were performed by placing plates in constant light at 22°C, and germination was scored after 7 days. Dormancy analysis was followed by stratification of non-germinated seeds to remove supposedly dead seeds from the analysis. Seed stratification was performed for 7 days at 4°C.

Seed size measurements

Seed size analysis was primarily performed using the Boxeed robot with its two modes. In seed-sowing mode, two photos of the seed (with 90° angle rotation) were taken before placing the seed on the plate. Seed parameters, including seed size, were obtained using Boxeed software. An average of two measurements was used to calculate seed size by multiplying it by the squared value of the coefficient (mm/px). In seed-sorting mode, two seed photos were taken to estimate the size of the seed before placing it into a separate 1.5-ml tube. The program's first run was performed to obtain the seed size distribution of the assayed pool, and seeds were then sorted into separate tubes on the basis of their size (Supplemental Table 1). Where indicated, additional analysis of seed size was performed using a stereomicroscope (Leica M205FA): dry seeds were placed on dry paper, and photos were taken before the seeds were soaked. Seed sizes were calculated using ImageJ software (Schneider et al., 2012). For analysis of seed sizes throughout the *Arabidopsis* life span, we used a Leica stereomicroscope equipped with an automated stage and PartSeg software (Bokota et al., 2021).

RNA-seq analysis

Four 100-seed biological replicates were used for transcriptomic analysis of small, medium, and large dry Col-0 seeds. Parameters of Boxeed seed sorting are provided in Supplemental Table 1. RNA was isolated using a standard protocol (Meng and Feldman, 2010), treated with Turbo DNase (Thermo Fisher), and 500 ng of RNA was used for reverse transcription with 50 mM library-specific barcoded and UMI (unique molecular identifier)-containing oligo(dT) primers (Supplemental Table 2) and SuperScript III (Thermo Fisher). Libraries for 3′ RNA-seq of seed pools and for single-seed RNA-seq were prepared as described previously (Krzyszton et al., 2022). In single-seed RNA-seq, each condition consisted of 96 seeds and was divided into three Illumina barcoded pools with 32 single seeds analyzed in each. Libraries were sequenced on the Illumina NextSeq 500 system using paired-end mode to obtain 21-nt R1 (containing barcode and UMI) and 55-nt R2 (containing mRNA sequence). Bioinformatic analysis of libraries for single-seed RNA-seq was performed as described in detail in our earlier work establishing the single-seed RNA-seq method (Hao et al., 2021; Krzyszton et al., 2022) and on the accompanying GitHub web page (https://github.com/mk1859/single_seed). We excluded low expressed genes with <1 read per seed on average. We also removed genes whose read number was correlated (Pearson correlation >0.3) with the number of intergenic reads in any condition, as such reads may not reflect accurate expression levels (Krzyszton et al., 2022). The remaining 6659 genes were analyzed with Seurat software and sctransform normalization (Hafemeister and Satija, 2019; Hao et al., 2021). Analysis of seed-pool 3′ RNA-seq data was also performed as described previously (Krzyszton et al., 2022) with a few changes. In brief, libraries were analyzed as described for single-seed RNA-seq to obtain read counts for genes. To obtain the deduplicated mapped reads shown in Figure 3A and 3B, the pipeline described for analysis of Col-0 and *dog1-4* mutant seed pools was used, as it involves the use of UMI-tools to produce deduplicated bam files (Smith et al., 2016). GO term analysis was performed using the gprofiler2 R package (Raudvere et al., 2019). The code used for analysis of RNA-seq datasets in this work is available at https://github.com/mk1859/seed_size.

RT–qPCR and smFISH

For RT–qPCR analysis, DNase-treated RNA was reverse transcribed using SuperScript III with a mixture of random and oligo(dT) primers. cDNA was diluted and used as a template in the qPCR reaction with LightCycler 480 SYBR Green I Master mix and the Roche LightCycler 480 instrument (primer sequences are provided in Supplemental Table 2). For fold change calculation, expression of the transcript was first normalized to mRNA of the *UBC21* (*AT5G25760*) reference gene and then to the average value calculated for large or small seeds. smFISH analysis was performed as described previously (Montez et al., 2023).

DATA AND CODE AVAILABILITY

The datasets generated and analyzed during the current study are available in the GEO repository under accession number GSE203310. Single-seed RNA-seq analysis for small and large seeds was performed together with analysis of Col-0 and the *dog1-4* mutant, and these data are available under accession number GSE185033.

SUPPLEMENTAL INFORMATION

Supplemental information is available at *Plant Communications Online*.

FUNDING

This work was funded by the Foundation for Polish Science (TEAM POIR.04.04.00-00-3C97/16-00) and by a National Science Centre, Poland grant (SONATA BIS UMO-2018/30/E/NZ1/00354) to S.S. M.K. was supported by a National Science Centre, Poland grant (OPUS UMO-2021/41/B/NZ3/02605). T.D. was supported by Statutory city of Brno and the JIC innovation agency program Prototypuj a ověřuj. D.P. and G.B. were supported by a Polish National Science Centre grant (2020/37/B/NZ2/03757). The funding bodies had no role in the design of the study, the collection, analysis, and interpretation of the data, or the writing of the manuscript.

AUTHOR CONTRIBUTIONS

M.K. performed RNA-seq experiments and analyzed all sequencing data. S.P.S. performed RT–qPCR analysis. V.H.M. performed the smFISH experiment. T.D., S.P.S., M.K., V.H.M., C.W., and K.M. performed seed size analysis and physiological tests. P.B. and B.G. developed PartSeg software and its plugin for seed size analysis during plant development and for smFISH analysis. S.S. supervised all experiments. S.S. and M.K. wrote the manuscript. All authors read and approved the final manuscript.

ACKNOWLEDGMENTS

We thank Vladyslava Liudkovska, Joanna Kufel, and Tomasz Turowski for their critical reading of the manuscript. T.D. is the founder and owner of Labdeers Ltd., which produces the Boxeed robot. No other conflicts of interest are declared.

Received: July 21, 2023
Revised: October 3, 2023
Accepted: October 11, 2023
Published: October 12, 2023

REFERENCES

Abley, K., Formosa-Jordan, P., Tavares, H., Chan, E.Y., Afsharinafar, M., Leyser, O., and Locke, J.C. (2021). An ABA-GA bistable switch can account for natural variation in the variability of Arabidopsis seed germination time. Elife **10**, e59485.

Baskin, C.C., and Baskin, J.M. (2014). Variation in Seed Dormancy and Germination within and between Individuals and Populations of a Species. Seeds, 277–373.

Batlla, D., Malavert, C., Farnocchia, R.B.F., Footitt, S., Benech-Arnold, R.L., and Finch-Savage, W.E. (2022). A quantitative analysis of temperature-dependent seasonal dormancy cycling in buried *Arabidopsis thaliana* seeds can predict seedling emergence in a global warming scenario. J. Exp. Bot. **73**:2454–2468.

Bentsink, L., Jowett, J., Hanhart, C.J., and Koornneef, M. (2006). Cloning of DOG1, a quantitative trait locus controlling seed dormancy in Arabidopsis. Proc. Natl. Acad. Sci. USA **103**:17042–17047.

Bhaskara, G.B., Nguyen, T.T., and Verslues, P.E. (2012). Unique drought resistance functions of the *Highly ABA-Induced* clade A protein phosphatase 2Cs. Plant Physiol. **160**:379–395.

Bokota, G., Sroka, J., Basu, S., Das, N., Trzaskoma, P., Yushkevich, Y., Grabowska, A., Magalska, A., and Plewczynski, D. (2021). PartSeg: a tool for quantitative feature extraction from 3D microscopy images for dummies. BMC Bioinf. **22**:72.

Bradford, K.J. (2018). Interpreting biological variation: seeds, populations and sensitivity thresholds. Seed Sci. Res. **28**:158–167.

Buijs, G. (2020). A Perspective on Secondary Seed Dormancy in Arabidopsis thaliana. Plants **9**:749.

Buijs, G., Vogelzang, A., Nijveen, H., and Bentsink, L. (2020). Dormancy cycling: translation-related transcripts are the main difference between dormant and non-dormant seeds in the field. Plant J. **102**:327–339.

Burghardt, L.T., Edwards, B.R., and Donohue, K. (2016). Multiple paths to similar germination behavior in *Arabidopsis thaliana*. New Phytol. **209**:1301–1312.

Chauffour, F., Bailly, M., Perreau, F., Cueff, G., Suzuki, H., Collet, B., Frey, A., Clément, G., Soubigou-Taconnat, L., Balliau, T., et al. (2019). Multi-omics analysis reveals sequential roles for ABA during seed maturation. Plant Physiol. **180**:1198–1218.

Cheng, L., Shafiq, S., Xu, W., and Sun, Q. (2018). EARLY FLOWERING IN SHORT DAYS (EFS) regulates the seed size in Arabidopsis. Sci. China Life Sci. **61**:214–224.

Chiang, G.C.K., Bartsch, M., Barua, D., Nakabayashi, K., Debieu, M., Kronholm, I., Koornneef, M., Soppe, W.J.J., Donohue, K., and De MEAUX, J. (2011). DOG1 expression is predicted by the seed-maturation environment and contributes to geographical variation in germination in Arabidopsis thaliana. Mol. Ecol. **20**:3336–3349.

Cyrek, M., Fedak, H., Ciesielski, A., Guo, Y., Sliwa, A., Brzezniak, L., Krzyczmonik, K., Pietras, Z., Kaczanowski, S., Liu, F., and Swiezewski, S. (2016). Seed dormancy in Arabidopsis is controlled by alternative polyadenylation of DOG1. Plant Physiol. **170**:947–955.

Dekkers, B.J.W., He, H., Hanson, J., Willems, L.A.J., Jamar, D.C.L., Cueff, G., Rajjou, L., Hilhorst, H.W.M., and Bentsink, L. (2016). The Arabidopsis *DELAY OF GERMINATION 1* gene affects *ABSCISIC ACID INSENSITIVE 5 (ABI5)* expression and genetically interacts with *ABI3* during Arabidopsis seed development. Plant J. **85**:451–465.

Dolata, J., Guo, Y., Kołowerzo, A., Smoliński, D., Brzyżek, G., Jarmołowski, A., and Świeżewski, S. (2015). NTR1 is required for transcription elongation checkpoints at alternative exons in Arabidopsis. EMBO J. **34**:544–558.

Doughty, J., Aljabri, M., and Scott, R.J. (2014). Flavonoids and the regulation of seed size in *Arabidopsis*. Biochem. Soc. Trans. **42**:364–369.

Elwell, A.L., Gronwall, D.S., Miller, N.D., Spalding, E.P., and Brooks, T.L.D. (2011). Separating parental environment from seed size effects on next generation growth and development in Arabidopsis: Effects of seed size and parental environment on Arabidopsis growth. Plant Cell Environ. **34**:291–301.

Fedak, H., Palusinska, M., Krzyczmonik, K., Brzezniak, L., Yatusevich, R., Pietras, Z., Kaczanowski, S., and Swiezewski, S. (2016). Control of seed dormancy in *Arabidopsis* by a *cis* -acting noncoding antisense transcript. Proc. Natl. Acad. Sci. USA **113**:E7846–E7855.

Footitt, S., Ölçer-Footitt, H., Hambidge, A.J., and Finch-Savage, W.E. (2017). A laboratory simulation of *Arabidopsis* seed dormancy cycling provides new insight into its regulation by clock genes and the dormancy-related genes *DOG1, MFT, CIPK23* and *PHYA*: Dormancy cycling with mutants. Plant Cell Environ. **40**:1474–1486.

Footitt, S., Walley, P.G., Lynn, J.R., Hambidge, A.J., Penfield, S., and Finch-Savage, W.E. (2020). Trait analysis reveals DOG1 determines initial depth of seed dormancy, but not changes during dormancy cycling that result in seedling emergence timing. New Phytol. **225**:2035–2047.

Fujii, H., Verslues, P.E., and Zhu, J.-K. (2011). *Arabidopsis* decuple mutant reveals the importance of SnRK2 kinases in osmotic stress responses in vivo. Proc. Natl. Acad. Sci. USA **108**:1717–1722.

Garcia, D., Saingery, V., Chambrier, P., Mayer, U., Jürgens, G., and Berger, F. (2003). Arabidopsis *haiku* mutants reveal new controls of seed size by endosperm. Plant Physiol. **131**:1661–1670.

Gianella, M., Bradford, K.J., and Guzzon, F. (2021). Ecological, (epi) genetic and physiological aspects of bet-hedging in angiosperms. Plant Reprod. **34**:21–36.

Gnan, S., Priest, A., and Kover, P.X. (2014). The genetic basis of natural variation in seed size and seed number and their trade-off using *Arabidopsis thaliana* MAGIC lines. Genetics **198**:1751–1758.

Graeber, K., Linkies, A., Steinbrecher, T., Mummenhoff, K., Tarkowská, D., Turečková, V., Ignatz, M., Sperber, K., Voegele, A., de Jong, H., et al. (2014). *DELAY OF GERMINATION 1* mediates a conserved coat-dormancy mechanism for the temperature- and gibberellin-dependent control of seed germination. Proc. Natl. Acad. Sci. USA **111**:E3571–E3580.

Gulden, R.H., Thomas, A.G., and Shirtliffe, S.J. (2004). Relative contribution of genotype, seed size and environment to secondary seed dormancy potential in Canadian spring oilseed rape (Brassica napus). Weed Res. **44**:97–106.

Hafemeister, C., and Satija, R. (2019). Normalization and variance stabilization of single-cell RNA-seq data using regularized negative binomial regression. Genome Biol. **20**:296.

Hao, Y., Hao, S., Andersen-Nissen, E., Mauck, W.M., Zheng, S., Butler, A., Lee, M.J., Wilk, A.J., Darby, C., Zager, M., et al. (2021). Integrated analysis of multimodal single-cell data. Cell **184**:3573–3587.e29.

Herridge, R.P., Day, R.C., Baldwin, S., and Macknight, R.C. (2011). Rapid analysis of seed size in Arabidopsis for mutant and QTL discovery. Plant Methods **7**:3.

Ibarra, S.E., Tognacca, R.S., Dave, A., Graham, I.A., Sánchez, R.A., and Botto, J.F. (2016). Molecular mechanisms underlying the entrance in secondary dormancy of *Arabidopsis* seeds: Mechanisms underlying secondary dormancy. Plant Cell Environ. **39**:213–221.

Jiang, W.-B., Huang, H.-Y., Hu, Y.-W., Zhu, S.-W., Wang, Z.-Y., and Lin, W.-H. (2013). Brassinosteroid regulates seed size and shape in Arabidopsis. Plant Physiol. **162**:1965–1977.

Joosen, R.V.L., Arends, D., Willems, L.A.J., Ligterink, W., Jansen, R.C., and Hilhorst, H.W.M. (2012). Visualizing the genetic landscape of Arabidopsis seed performance. Plant Physiol. **158**:570–589.

Jurado, E., and Flores, J. (2005). Is seed dormancy under environmental control or bound to plant traits? J. Veg. Sci. **16**:559–564.

Kanno, Y., Jikumaru, Y., Hanada, A., Nambara, E., Abrams, S.R., Kamiya, Y., and Seo, M. (2010). Comprehensive hormone profiling in developing Arabidopsis seeds: examination of the site of ABA biosynthesis, ABA transport and hormone interactions. Plant Cell Physiol. **51**:1988–2001.

Kendall, S.L., Hellwege, A., Marriot, P., Whalley, C., Graham, I.A., and Penfield, S. (2011). Induction of Dormancy in Arabidopsis Summer Annuals Requires Parallel Regulation of DOG1 and Hormone Metabolism by Low Temperature and CBF Transcription Factors. Plant Cell **23**:2568–2580. https://doi.org/10.1105/tpc.111.087643.

Kerdaffrec, E., Filiault, D.L., Korte, A., Sasaki, E., Nizhynska, V., Seren, Ü., and Nordborg, M. (2016). Multiple alleles at a single locus control seed dormancy in Swedish Arabidopsis. Elife **5**, e22502.

Krannitz, P.G., Aarssen, L.W., and Dow, J.M. (1991). The effect of genetically based differences in seed size on seedling survival in Arabidopsis thaliana (Brassicaceae). Am. J. Bot. **78**:446–450.

Krzyszton, M., Yatusevich, R., Wrona, M., Sacharowski, S.P., Adamska, D., and Swiezewski, S. (2022). Single seeds exhibit transcriptional heterogeneity during secondary dormancy induction. Plant Physiol. **190**:211–225.

Kushiro, T., Okamoto, M., Nakabayashi, K., Yamagishi, K., Kitamura, S., Asami, T., Hirai, N., Koshiba, T., Kamiya, Y., and Nambara, E. (2004). The Arabidopsis cytochrome P450 CYP707A encodes ABA 8'-hydroxylases: key enzymes in ABA catabolism. EMBO J. **23**:1647–1656.

Larios, E., Búrquez, A., Becerra, J.X., and Lawrence Venable, D. (2014). Natural selection on seed size through the life cycle of a desert annual plant. Ecology **95**:3213–3220.

Leon-Kloosterziel, K.M., Keijzer, C.J., and Koornneef, M. (1994). A seed shape mutant of Arabidopsis that is affected in integument development. Plant Cell **6**:385–392.

Li, N., and Li, Y. (2016). Signaling pathways of seed size control in plants. Curr. Opin. Plant Biol. **33**:23–32.

Li, C., Chen, B., and Yu, H. (2022). Splicing-mediated activation of SHAGGY-like kinases underpinning carbon partitioning in Arabidopsis seeds. Plant Cell **34**:2730–2746.

Liu, Y., Walck, J.L., and El-Kassaby, Y.A. (2017). Roles of the Environment in Plant Life-History Trade-offs. In Advances in Seed Biology, J.C. Jimenez-Lopez, ed. (InTech).

Liu, R., Wang, L., Tanveer, M., and Song, J. (2018). Seed heteromorphism: An important adaptation of halophytes for habitat heterogeneity. Front. Plant Sci. **9**:1515.

Liu, Z., Zheng, L., Pu, L., Ma, X., Wang, X., Wu, Y., Ming, H., Wang, Q., and Zhang, G. (2020). ENO2 affects the seed size and weight by adjusting cytokinin content and forming ENO2-bZIP75 complex in Arabidopsis thaliana. Front. Plant Sci. **11**, 574316.

Liu, H., Luo, Q., Tan, C., Song, J., Zhang, T., and Men, S. (2023). Biosynthesis- and transport-mediated dynamic auxin distribution during seed development controls seed size in Arabidopsis. Plant J. **113**:1259–1277.

Matilla, A., Gallardo, M., and Puga-Hermida, M.I. (2005). Structural, physiological and molecular aspects of heterogeneity in seeds: a review. Seed Sci. Res. **15**:63–76.

Meng, L., and Feldman, L. (2010). A rapid TRIzol-based two-step method for DNA-free RNA extraction from Arabidopsis siliques and dry seeds. Biotechnol. J. **5**:183–186.

Mira, S., Veiga-Barbosa, L., and Pérez-García, F. (2019). Seed dormancy and longevity variability of *Hirschfeldia incana* L. during storage. Seed Sci. Res. **29**:97–103.

Montez, M., Majchrowska, M., Krzyszton, M., Bokota, G., Sacharowski, S., Wrona, M., Yatusevich, R., Massana, F., Plewczynski, D., and Swiezewski, S. (2023). Promoter-pervasive transcription causes RNA polymerase II pausing to boost DOG1 expression in response to salt. EMBO J. **42**:e112443.

Nakabayashi, K., Bartsch, M., Xiang, Y., Miatton, E., Pellengahr, S., Yano, R., Seo, M., and Soppe, W.J.J. (2012). The time required for dormancy release in *Arabidopsis* is determined by DELAY OF GERMINATION1 protein levels in freshly harvested seeds. Plant Cell **24**:2826–2838.

Née, G., Kramer, K., Nakabayashi, K., Yuan, B., Xiang, Y., Miatton, E., Finkemeier, I., and Soppe, W.J.J. (2017). DELAY OF GERMINATION1 requires PP2C phosphatases of the ABA signalling pathway to control seed dormancy. Nat. Commun. **8**:72.

Nemati, I., Sedghi, M., Hosseini Salekdeh, G., Tavakkol Afshari, R., Naghavi, M.R., and Gholizadeh, S. (2022). DELAY OF GERMINATION 1 (DOG1) regulates dormancy in dimorphic seeds of Xanthium strumarium. Funct. Plant Biol. **49**:742–758.

Nishimura, N., Yoshida, T., Kitahata, N., Asami, T., Shinozaki, K., and Hirayama, T. (2007). ABA-Hypersensitive Germination1 encodes a protein phosphatase 2C, an essential component of abscisic acid signaling in Arabidopsis seed: Arabidopsis ABA-hypersensitive mutant. Plant J. **50**:935–949.

Nonogaki, H. (2019). Seed germination and dormancy: The classic story, new puzzles, and evolution. J. Integr. Plant Biol. **61**:541–563.

Orozco-Arroyo, G., Paolo, D., Ezquer, I., and Colombo, L. (2015). Networks controlling seed size in Arabidopsis. Plant Reprod. **28**:17–32.

Raudvere, U., Kolberg, L., Kuzmin, I., Arak, T., Adler, P., Peterson, H., and Vilo, J. (2019). g:Profiler: a web server for functional enrichment analysis and conversions of gene lists. Nucleic Acids Res. **47**:W191–W198.

Ren, D., Wang, X., Yang, M., Yang, L., He, G., and Deng, X.W. (2019). A new regulator of seed size control in Arabidopsis identified by a genome-wide association study. New Phytol. **222**:895–906.

Riefler, M., Novak, O., Strnad, M., and Schmülling, T. (2005). *Arabidopsis* cytokinin receptor mutants reveal functions in shoot growth, leaf senescence, seed size, germination, root development, and cytokinin metabolism. Plant Cell **18**:40–54.

Robert, C., Noriega, A., Tocino, Á., and Cervantes, E. (2008). Morphological analysis of seed shape in Arabidopsis thaliana reveals altered polarity in mutants of the ethylene signaling pathway. J. Plant Physiol. **165**:911–919.

Rubio De Casas, R., Willis, C.G., Pearse, W.D., Baskin, C.C., Baskin, J.M., and Cavender-Bares, J. (2017). Global biogeography of seed dormancy is determined by seasonality and seed size: a case study in the legumes. New Phytol. **214**:1527–1536.

Sall, K., Dekkers, B.J.W., Nonogaki, M., Katsuragawa, Y., Koyari, R., Hendrix, D., Willems, L.A.J., Bentsink, L., and Nonogaki, H. (2019). DELAY OF GERMINATION1 - LIKE 4 acts as an inducer of seed reserve accumulation. Plant J. **100**:7–19.

Schneider, C.A., Rasband, W.S., and Eliceiri, K.W. (2012). NIH Image to ImageJ: 25 years of image analysis. Nat. Methods **9**:671–675.

Smith, T., Heger, A., and Sudbery, I. (2016). UMI-tools: Modelling sequencing errors in Unique Molecular Identifiers to improve quantification accuracy. Genome Res. **27**:491–499.

Soppe, W.J.J., and Bentsink, L. (2020). Seed dormancy back on track; its definition and regulation by DOG1. New Phytol. **228**:816–819.

Venable, D.L., and Brown, J.S. (1988). The selective interactions of dispersal, dormancy, and seed size as adaptations for reducing risk in variable environments. Am. Nat. **131**:360–384.

Vidigal, D.S., Marques, A.C.S.S., Willems, L.A.J., Buijs, G., Méndez-Vigo, B., Hilhorst, H.W.M., Bentsink, L., Picó, F.X., and Alonso-Blanco, C. (2016). Altitudinal and climatic associations of seed dormancy and flowering traits evidence adaptation of annual life cycle timing in *Arabidopsis thaliana*: Climatic adaptation of annual life cycles. Plant Cell Environ. **39**:1737–1748.

Volis, S. (2016). Seed heteromorphism in Triticum dicoccoides: association between seed positions within a dispersal unit and dormancy. Oecologia **181**:401–412.

Volis, S., and Bohrer, G. (2013). Joint evolution of seed traits along an aridity gradient: seed size and dormancy are not two substitutable evolutionary traits in temporally heterogeneous environment. New Phytol. **197**:655–667.

Xi, W., Liu, C., Hou, X., and Yu, H. (2010). *MOTHER OF FT AND TFL1* regulates seed germination through a negative feedback loop modulating ABA signaling in *Arabidopsis*. Plant Cell **22**:1733–1748.

Xiang, Y., Nakabayashi, K., Ding, J., He, F., Bentsink, L., and Soppe, W.J.J. (2014). *REDUCED DORMANCY5* encodes a protein phosphatase 2C that is required for seed dormancy in *Arabidopsis*. Plant Cell **26**:4362–4375.

Xu, Y., Zhao, Y., Duan, H., Sui, N., Yuan, F., and Song, J. (2017). Transcriptomic profiling of genes in matured dimorphic seeds of euhalophyte Suaeda salsa. BMC Genom. **18**:727.

Yatusevich, R., Fedak, H., Ciesielski, A., Krzyczmonik, K., Kulik, A., Dobrowolska, G., and Swiezewski, S. (2017). Antisense transcription represses *Arabidopsis* seed dormancy QTL *DOG 1* to regulate drought tolerance. EMBO Rep. **18**:2186–2196.

Yoshida, T., Nishimura, N., Kitahata, N., Kuromori, T., Ito, T., Asami, T., Shinozaki, K., and Hirayama, T. (2006). ABA-hypersensitive germination3 encodes a protein phosphatase 2C (AtPP2CA) that strongly regulates abscisic acid signaling during germination

among Arabidopsis protein phosphatase 2Cs. Plant Physiol. **140**:115–126.

Yu, B., He, X., Tang, Y., Chen, Z., Zhou, L., Li, X., Zhang, C., Huang, X., Yang, Y., Zhang, W., et al. (2023). Photoperiod controls plant seed size in a CONSTANS-dependent manner. Nat. Plants **9**:343–354.

Zhao, H., Nie, K., Zhou, H., Yan, X., Zhan, Q., Zheng, Y., and Song, C.P. (2020). ABI5 modulates seed germination via feedback regulation of the expression of the *PYR/PYL/RCAR* ABA receptor genes. New Phytol. **228**:596–608.

Zhou, Y., Zhang, X., Kang, X., Zhao, X., Zhang, X., and Ni, M. (2009). SHORT HYPOCOTYL UNDER BLUE1 associates with *MINISEED3* and *HAIKU2* promoters in vivo to regulate *Arabidopsis* seed development. Plant Cell **21**:106–117.

Plant Communications, Volume *5*

Supplemental information

Dormancy heterogeneity among *Arabidopsis thaliana* seeds is linked to individual seed size

Michal Krzyszton, Sebastian P. Sacharowski, Veena Halale Manjunath, Katarzyna Muter, Grzegorz Bokota, Ce Wang, Dariusz Plewczyński, Tereza Dobisova, and Szymon Swiezewski

Supplemental Figures

Dormancy heterogeneity among *Arabidopsis thaliana* seeds is linked to individual seed size

Michal Krzyszton, Sebastian P. Sacharowski, Veena Halale Manjunath, Katarzyna Muter, Grzegorz Bokota, Ce Wang, Dariusz Plewczyński, Tereza Dobisova, Szymon Swiezewski

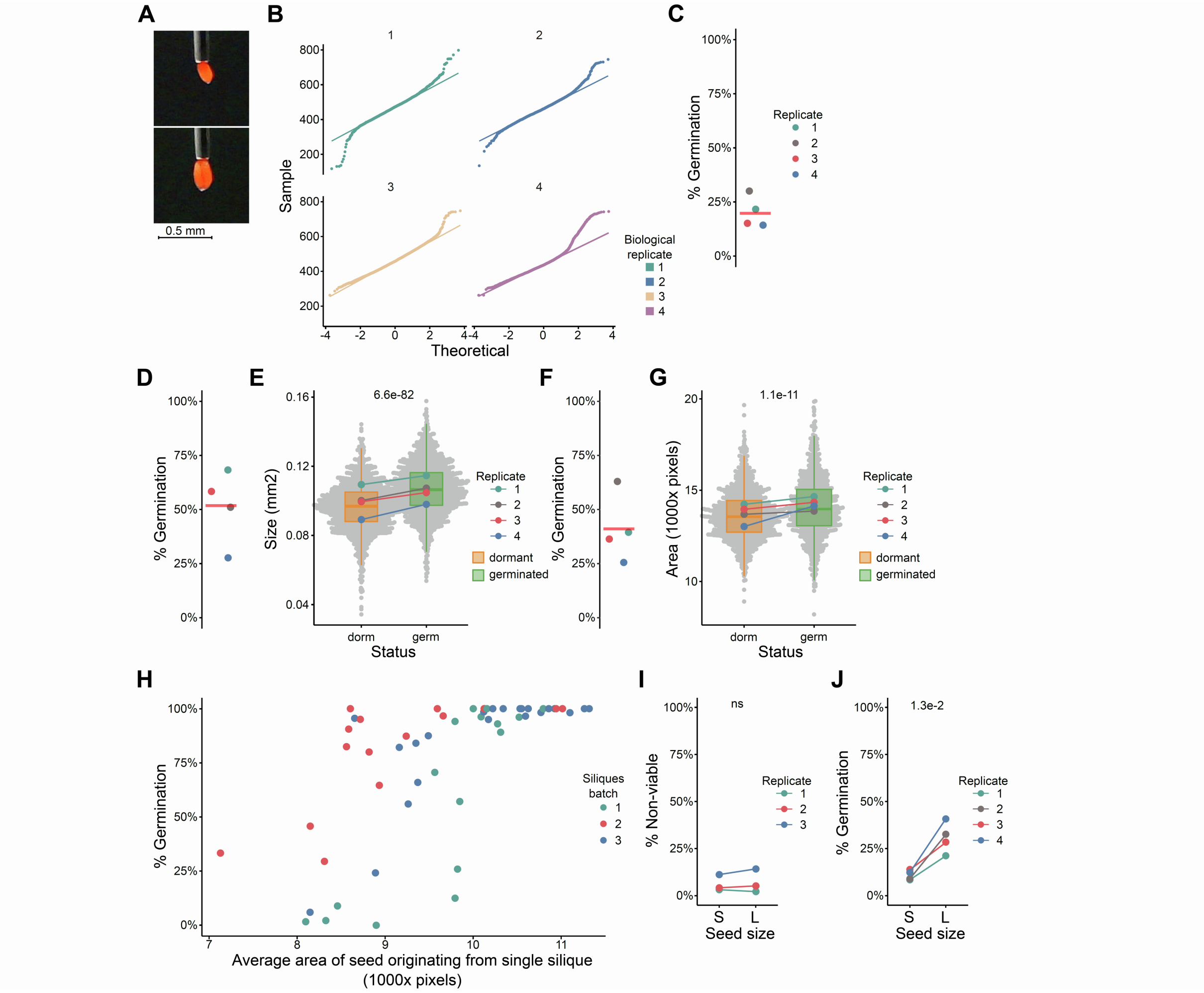

Supplemental Figure 1. Small seeds are more dormant. A) Examples of seeds' pictures obtained using the Boxeed robot. B) Deviations from the normal distribution of seed sizes in four seed pools from Figure 1A are shown using Q-Q plots. C) Germination of freshly harvested Col-0 seeds (n=320 to 607) was analyzed in four replicas. The red line denotes the mean value. D) Germination of after-ripened Col-0 seeds after 7 days of secondary dormancy induction (n=735 to 808) was analyzed in four replicas. The red line denotes the mean value. E) Size distribution of germinated (germ) and dormant (dorm) seeds from D. Mean values of seed sizes for dormant and germinated seeds are shown as points for each of the replicates. The p-value of the Wilcoxon rank-sum test for the comparison of seed sizes from all replicas is shown above the plot. F) Germination of Col-0 after-ripened seeds after 7 days of secondary dormancy induction. Four replicas of seed pools (n=450 to 799) were placed on the plate, photographed using a stereo microscope and seed sizes were assayed using ImageJ software. The red line denotes the mean value. G) Size distribution of germinated (germ) and dormant (dorm) seeds from F. Mean values of seed sizes for dormant and germinated seeds are shown as points for each of the replicas. The p-value of the Wilcoxon rank-sum test for seed sizes from all replicas is shown above the plot. H) Germination percentages of seeds from each silique were plotted against the average size of seeds from the siliques. I) Percentage of non-viable seeds after secondary dormancy induction treatment. Plates after scoring initial germination were placed for 7 days at 4°C after which seeds were again scored for seed germination. Seeds that did not germinate were considered non-viable. "ns" marks a lack of statistical significance (two-sided

paired t-test). J) The top 10% of the smallest and the largest seeds (n= 116-211) from four Col-0 after-ripened seed pools were sorted and tested for germination in the presence of 0.5 mM ABA. The p-value of the two-sided paired t-test is shown above the plot. Seeds were analyzed using a Boxeed robot. Seed germination was scored after 7 days.

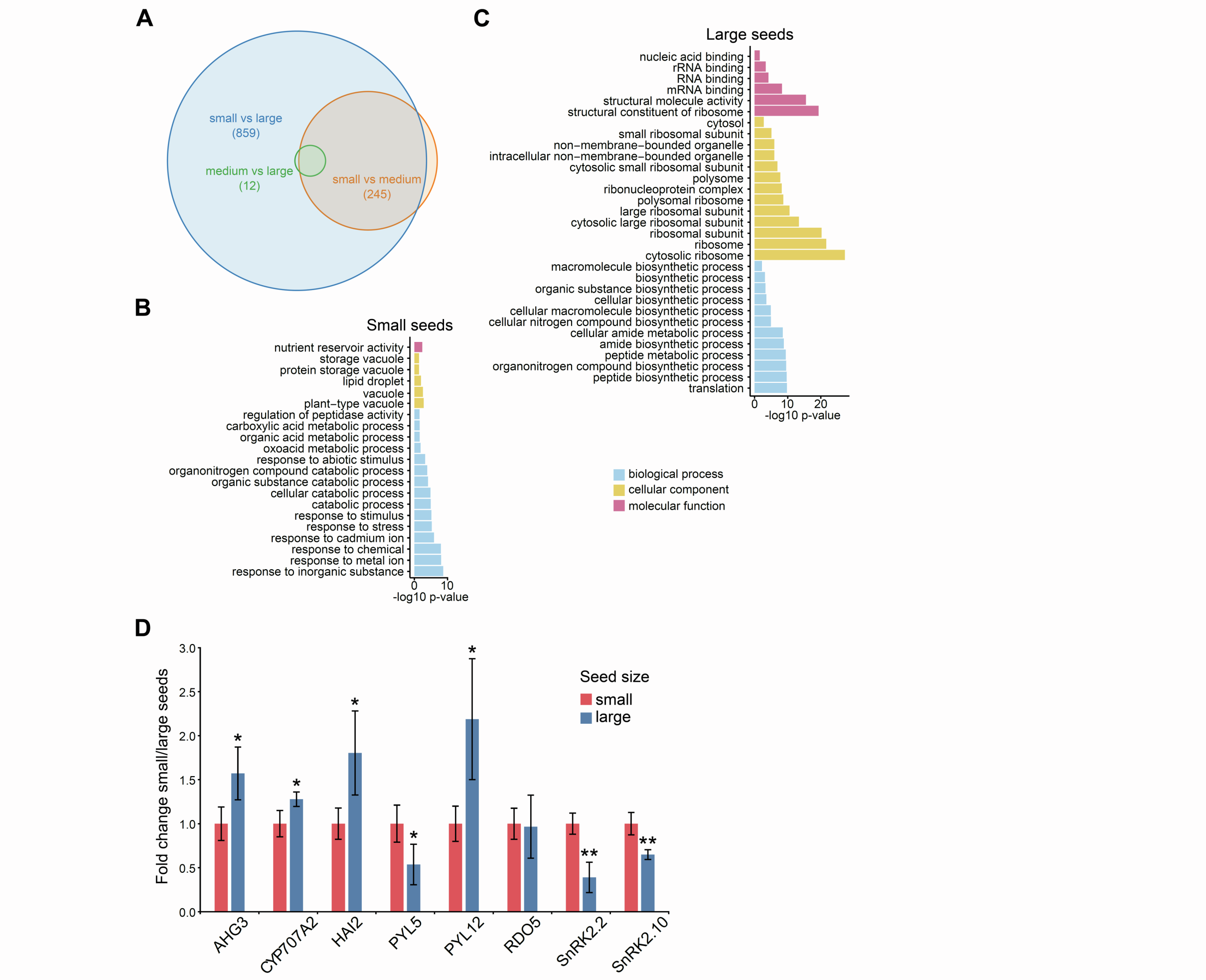

Supplemental Figure 2. Small and large seeds differ in transcriptome profiles. A) Differential gene expression analysis reveals that a similar set of genes is affected (DESeq2, padj < 0.05) when small, medium, and large seeds are compared. B) and C) GO terms enriched (gprofiler2) among genes with higher expression in small and large seeds, respectively. Colors show sources of gene ontologies. D) RT-qPCR analysis (four replicas of large and small Col-0 seeds) of selected genes with expression change in 3′RNA-seq. Fold change to the average value for small seeds is shown for each transcript and error bars show the standard deviation. Asterisks denote statistical significance (t-test): * p-value < 0.05 and ** p-value < 0.01. *UBC21* mRNA was used as a reference.

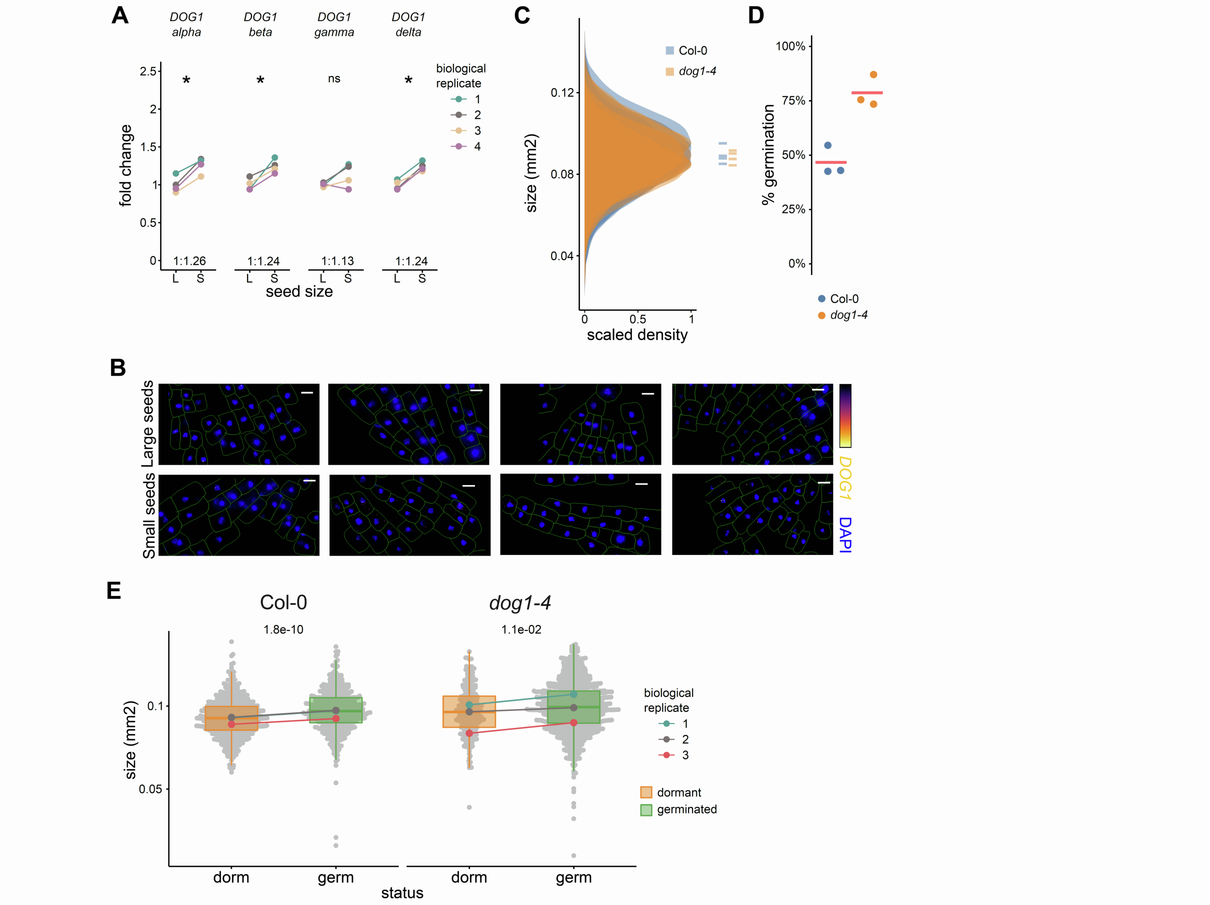

Supplemental Figure 3. *DOG1* expression differentiates seeds of different sizes. A) RT-qPCR analysis of long *DOG1* splicing isoforms (*alpha*, *beta*, *gamma*, *delta*) in four replicas of large and small Col-0 seeds. Fold change to the average value for large seeds is shown for each transcript. Asterisks denote p-value < 0.05, and "ns" marks a lack of statistical significance (paired t-test). Ratios of isoform levels are shown above the x-axis. *UBC21* mRNA was used as a reference. B) z-stack max-projection images of smFISH for *DOG1* mRNA. The "Inferno" colour scale is used for the intensity of fluorescence from Quasar670 fluorophore (*DOG1*). The blue colour shows *DAPI* fluorescence from (nuclei staining). The scale bar is 20 µm. C) Seeds' sizes of Col-0 and *dog1-4* mutant were obtained using the Boxeed robot. Median values of seeds' size for each tested seed pool are shown as a line on the right. 3000 seeds were analyzed for each pool. D) Col-0 and *dog1-4* mutant after-ripened seeds germination after 7 days of secondary dormancy induction (n=354 to 384). Red lines denote mean values. E) Size distribution of germinated (germ) and dormant (dor) seeds from D. Mean values of seed sizes for dormant and germinated seeds are shown as points for each of the replicas. The p-values of the Wilcoxon rank-sum test for comparison of seed sizes for all replicas are shown above the plots. Seeds were sown using a Boxeed robot and seeds' morphological parameters were obtained using Boxeed software.

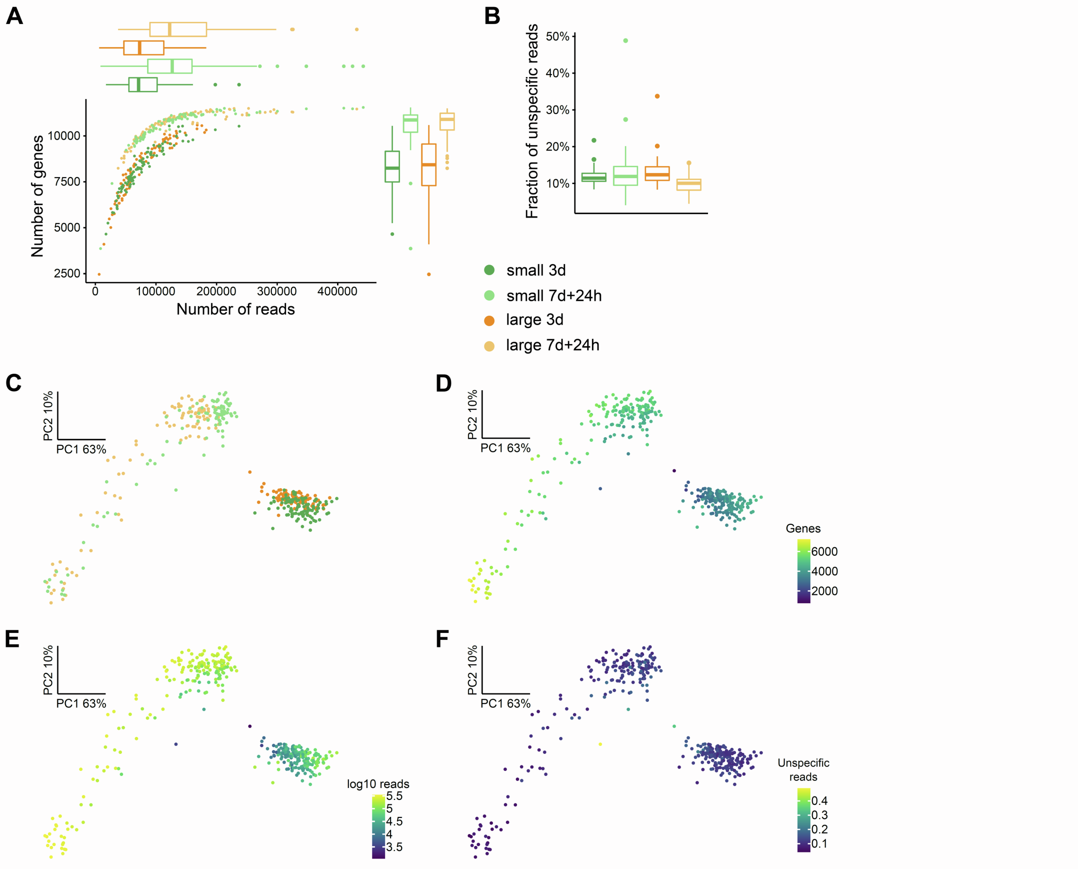

Supplemental Figure 4. Quality controls for single seed RNA-seq from small and large seeds experiment. A) Plot showing the number of reads sequenced and genes identified for each seed (n = 384). Boxplots above and on the right of the plot show the number of reads sequenced and genes identified respectively for each time point. Boxplot whiskers show a 1.5 interquartile range, outliers are marked with dots. B) Boxplots showing the fraction of unspecific reads for each seed in each condition. Boxplot whiskers show a 1.5 interquartile range, outliers are marked with dots. C) PCA plot of seeds' transcriptomes (Seurat with sctransform normalization). D-F) PCA plots with the number of sequenced reads, identified genes, and the fraction of intergenic reads.

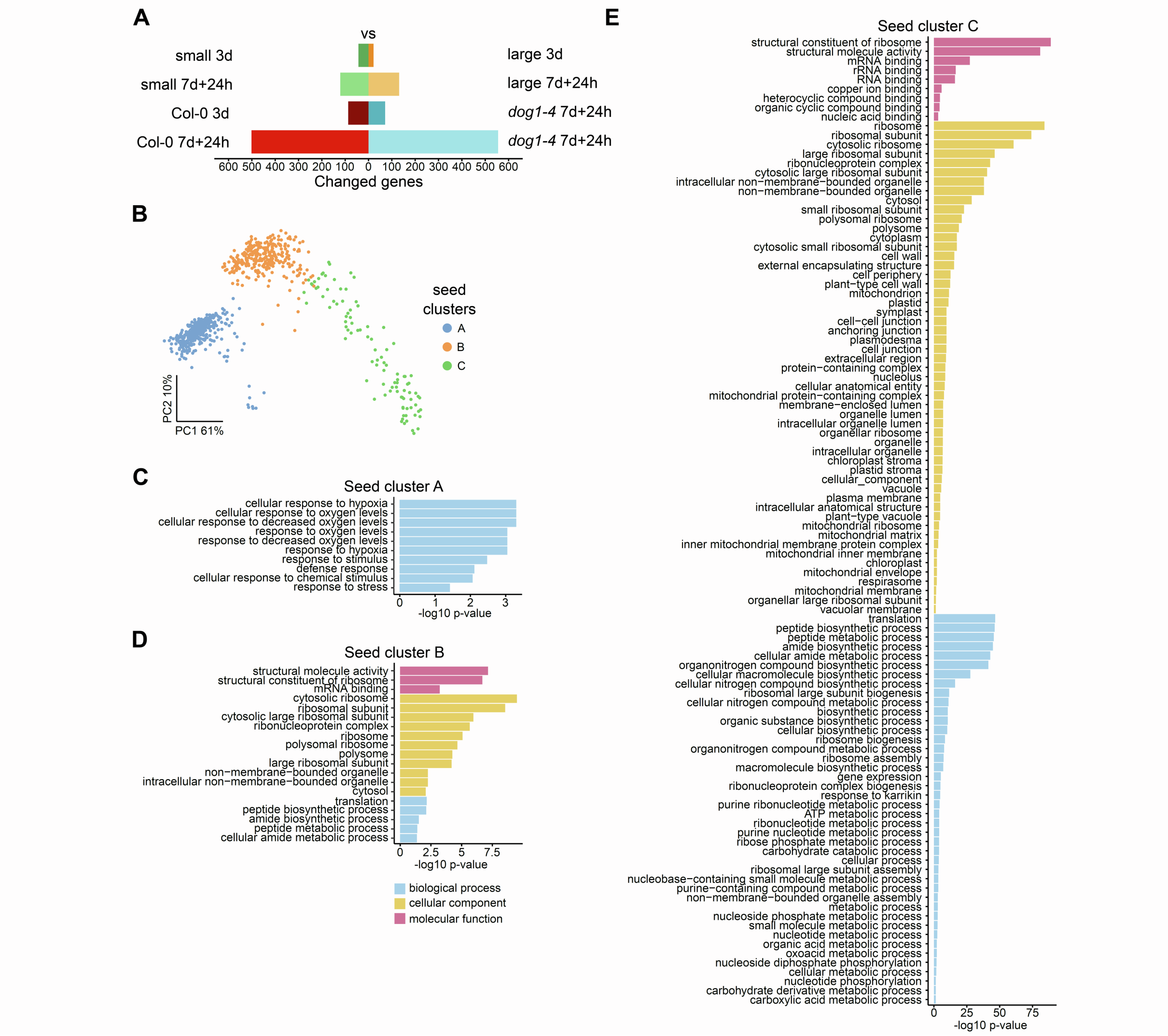

Supplemental Figure 5. The expression of translation-related genes is upregulated in the "tail" of the 7d+24h condition in the PCA plot. A) Number of genes identified as upregulated in seeds when different conditions were compared (Seurat FindMarkers Wilcoxon Rank Sum test; adjusted p-value < 0.05; $|\log_2 FC| > \log_2(1.2)$). B) PCA plot with three clusters of seeds identified by hierarchical clustering. C-E) GO terms enriched (gprofiler2) among genes whose expression is upregulated (Seurat FindMarkers Wilcoxon Rank Sum test; adjusted p-value < 0.05; $|\log_2 FC| > \log_2(1.5)$) in each seeds' clusters. Colors show sources of gene ontologies.

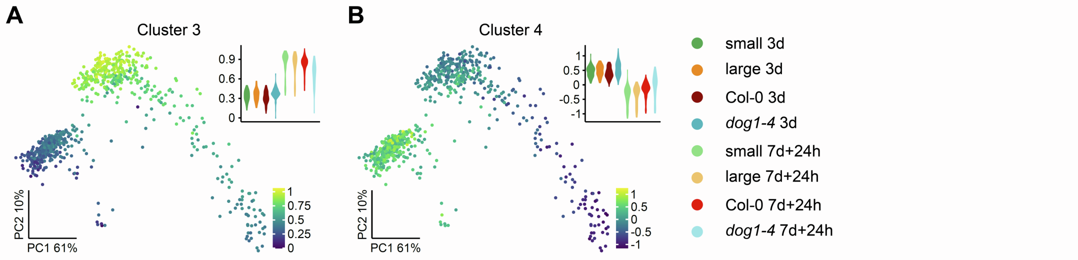

Supplemental Figure 6. Co-expressed gene groups. A-B) Gene expression signature values (mean normalized expression) of two other co-expressed gene groups overlaid on the PCA plot. Gene expression correlation among seeds was calculated (scran R package), and gene pairs with correlation > 0.5 were used for clustering (RBGL R package). Gene groups with at least 10 genes were kept. Identified groups were used to calculate signatures (Seurat AddModuleScore). Signature values spread for each condition are shown as a violin plot.

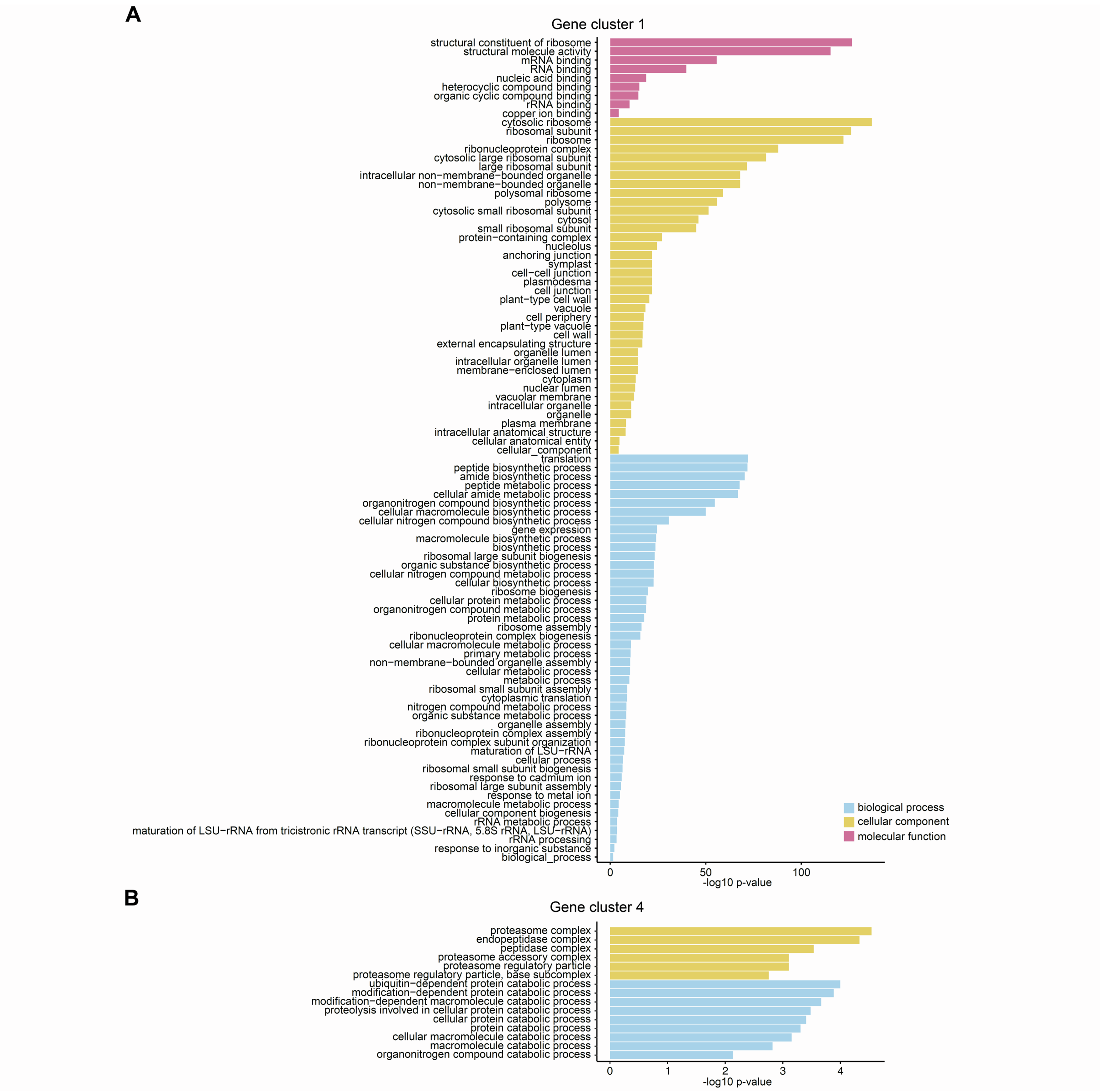

Supplemental Fig. S7. A-B) GO terms enriched (gprofiler2) among genes grouped in co-expressed cluster 1 (A; Figure 5A) and co-expressed cluster 4 (B; Supplemental Figure 6B). Colors show sources of gene ontologies.

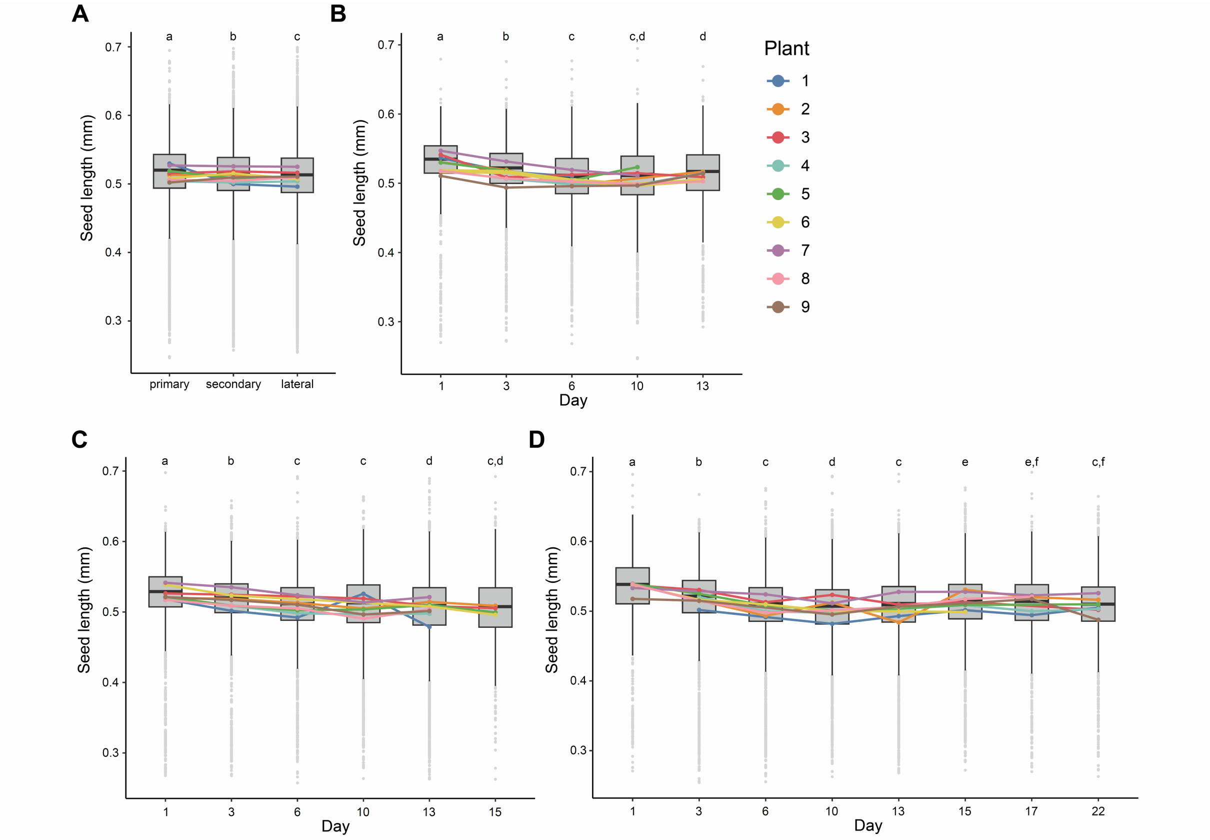

Supplemental Figure 8. Seed size slightly changes during the reproductive stage in Arabidopsis. A) Comparison of seed sizes collected from different parts of the plant (primary, secondary and lateral shoots). B) Comparison of seed sizes collected on different days from the primary shoot. C) Comparison of seed sizes collected on different days from secondary shoots. C) Comparison of seed sizes collected on different days from lateral shoots. Seeds were collected from nine plants. The letters above show the results of the pairwise Wilcoxon test (p-value < 0.001) performed for seeds pooled from all plants.
